# Neutrophil immunometabolism in ACLF and sepsis: mechanisms, dysfunction, and therapeutic opportunities

**DOI:** 10.3389/fimmu.2026.1828801

**Published:** 2026-05-22

**Authors:** Sonali Mukherjee, Takhellambam Malemnganba, Pragyan Acharya

**Affiliations:** Department of Biochemistry, All India Institute of Medical Sciences, New Delhi, India

**Keywords:** acute on chronic liver failure (ACLF), immune dysfunction, immune reprogramming, immunometabolism, inflammation, metabolic exhaustion, NEtosis, sepsis

## Abstract

Innate immune cells, undergo profound metabolic changes in critical illnesses. In both, acute-on-chronic liver failure (ACLF) and sepsis, these alterations underpin the paradoxical coexistence of hyperinflammation and immune dysfunction. Here, we present a comparative framework to examine how immune metabolic circuits are reshaped across these two syndromes. We focus primarily on neutrophil function while also considering contributions from other immune cell types, highlighting shared pathways, divergent mechanisms, and their clinical implications. We first delineate shared features of neutrophil activation in critical illness, including glycolysis-driven metabolic reprogramming, excessive reactive oxygen species (ROS) generation, and neutrophil extracellular trap (NET) formation, all processes that amplify tissue injury and propagate systemic inflammation. However, fundamental differences emerge in the baseline immune state, trajectory, and underlying immunometabolic programming of the two diseases. Sepsis arises as an acute insult in a previously homeostatic immune system, triggering a rapid transition from hyperactivation to mitochondrial dysfunction and eventual metabolic exhaustion. In contrast, ACLF develops on a background of chronic liver disease, where immune cells are already primed and metabolically stressed, resulting in a constrained and dysfunctional response from the outset. By placing ACLF and sepsis side by side, this review highlights the metabolic regulation of innate immunity, particularly neutrophils, as both a unifying principle and a disease-specific vulnerability. This comparative perspective deepens mechanistic understanding and provides a framework for precision immunometabolic interventions in critical illness.

## Introduction

Acute-on-chronic liver failure (ACLF) and sepsis are distinct but clinically convergent syndromes characterized by systemic inflammation, immune dysregulation, and high short-term mortality (1–3). Sepsis is defined as a life-threatening organ dysfunction caused by a dysregulated host response to infection, typically arising in a previously homeostatic immune system (1). In contrast, ACLF develops in patients with underlying chronic liver disease following an acute insult, and is characterized by rapid hepatic decompensation accompanied by extrahepatic organ failure (4). Several ACLF patients also have sepsis leading to a complex, heterogeneous etiology over an underlying chronic disease. Despite differences in etiology and baseline immune state, both syndromes share the paradoxical coexistence of hyperinflammation and impaired host defense, making them compelling models to study immune dysfunction in critical illness. In this review, we aim to compare neutrophil immunometabolism in ACLF and sepsis, highlighting both shared mechanisms and key differences, and discussing their implications for targeted therapeutic strategies.

Neutrophils, as rapid first responders of the innate immune system, are central to this paradox. They exhibit concurrent hyperactivation and functional paralysis in both conditions (5–7). Neutrophil effector functions such as chemotaxis, degranulation and pathogen clearacnce are now known to be closely regulated by cellular metabolic programs (8). Advances in immunometabolism have established glycolysis, mitochondrial function and lipid metabolism as key regulators of neutrophil outputs e.g. reactive oxygen species (ROS), neutrophil extracellular traps (NETs) and its lifespan (9–11).

In both ACLF and sepsis, neutrophils undergo significant metabolic reprogramming in response to the hostile systemic environment, marked by hypoxia, endotoxemia, and altered nutrient availability (12–14). These metabolic shifts sustain inflammatory responses but, ultimately drive impaired bacterial clearance, tissue injury, and immunoparalysis (15, 16).

Despite this progress, a unified framework linking neutrophil immunometabolism to disease pathogenesis remains lacking. This gap is particularly evident in ACLF, where mechanistic resolution at the level of neutrophils is limited compared to sepsis. Emerging transcriptomic and clinical data support a model of simultaneous inflammatory activation and immune dysfunction in ACLF, yet these insights derive largely from bulk or mixed immune cell analyses (17–21). A structured comparison with sepsis is therefore necessary to distinguish shared inflammatory programs from what may be disease-specific immunometabolic constraints.

Systemic inflammation is often used as a unifying term across diverse syndromes characterized by innate immune activation. However, the limited success of broadly applied anti-inflammatory therapies in conditions such as sepsis, together with growing evidence for heterogeneous immune and metabolic states revealed by transcriptomic and single-cell studies (6), suggests that uniform therapeutic approaches may fail to capture the biological complexity of these syndromes. This raises a fundamental question: to what extent are inflammation shared across diseases and how much is shaped by disease-specific immune–metabolic states? Can we harness this distinction for contribution to precision therapies?

Bridging these gaps in knowledge is imperative not only for mechanistic understanding but also for the identification of novel biomarkers and therapeutic opportunities that restore immune balance in inflammation-driven diseases. In this analytical review, we examine the evolving landscape of immunometabolism through the lens of two syndromes where innate immune activation significantly influences clinical trajectory: ACLF and sepsis. We integrate evidence from clinical cohorts, experimental models and systems-level analyses. We compare these two syndromes side by side, to identify shared mechanisms and disease-specific rewiring of innate immune responses, highlighting emerging opportunities to therapeutically targeting immunometabolic pathways in critical illness.

## 2 Immunopathology of critical illness through the lens of neutrophils

This section examines neutrophil dysfunction in ACLF and sepsis, focusing on shared effector programs and their disease-specific dysregulation.

Neutrophils mediate core immune functions such as chemotaxis, phagocytosis, degranulation (proteases, myeloperoxidase release), respiratory burst (ROS), cytokine secretion and neutrophil extracellular trap (NET) formation, mechanisms that are essential for pathogen clearance (5, 22, 23).

In critical illnesses such as sepsis and ACLF, neutrophils are simultaneously exposed to a combination of pro-inflammatory signals, including pathogen-associated molecular patterns (PAMPs) and danger-associated molecular patterns (DAMPs), which drive their activation and prolong their lifespan by inhibiting spontaneous apoptosis (7, 24). While this extended survival may initially enhance pathogen clearance, it may also cause excessive inflammation and collateral tissue damage, as activated neutrophils release reactive oxygen species (ROS), proteases, and neutrophil extracellular traps (NETs) (25, 26). Dysregulation of neutrophil death pathways, including apoptosis, necroptosis, and NETosis, has been linked to impaired bactericidal capacity and amplified inflammatory responses, both of which are hallmarks of organ injury in critical illness (27, 28). While essential for pathogen clearance, dysregulated neutrophil activation contributes directly to tissue injury and organ failure (29). These mechanisms are broadly conserved however, their regulation diverges between sepsis and ACLF.

### 2.1 Neutrophil dysfunction in sepsis

**Figure 1:**
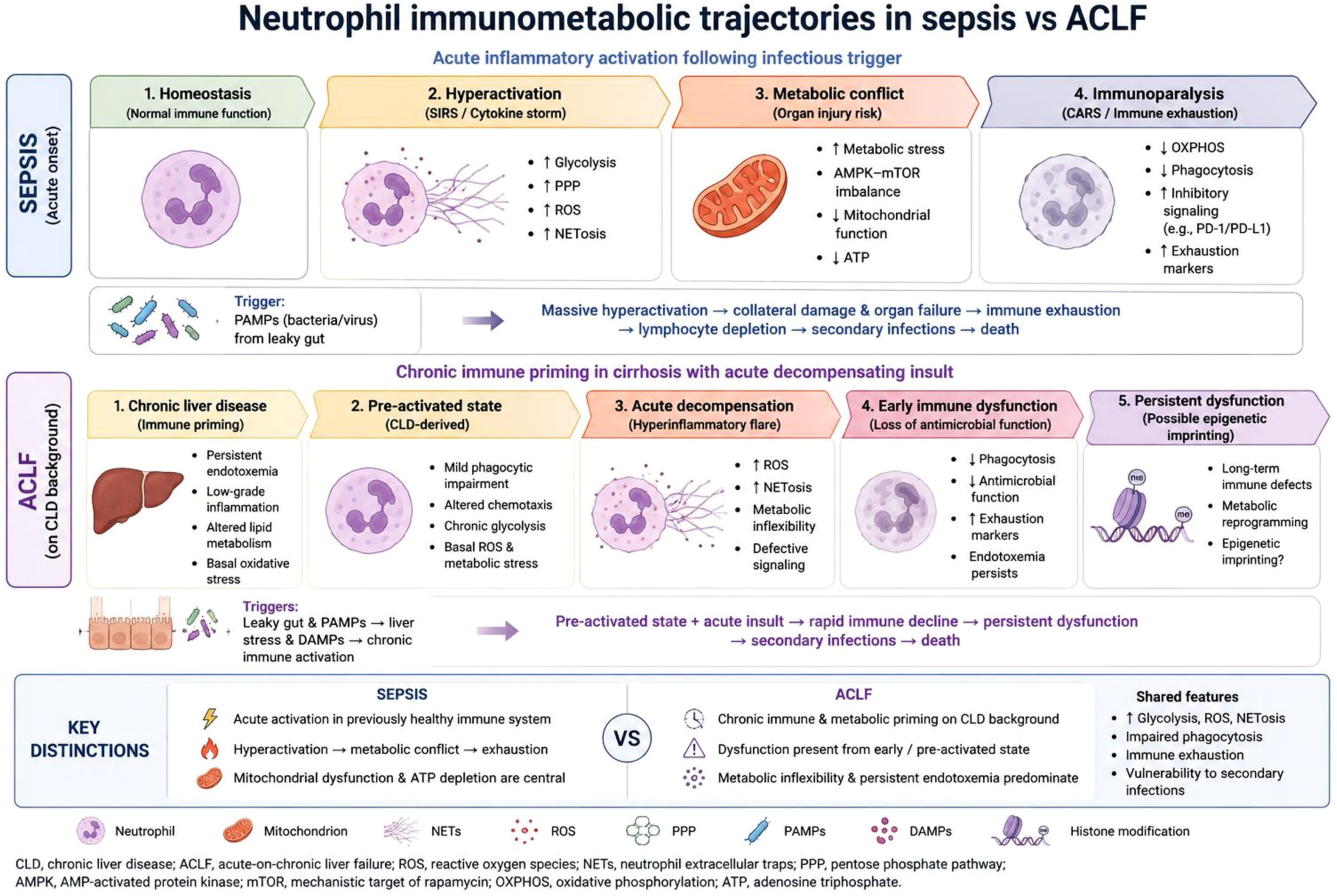
Distinct immunometabolic trajectories of neutrophils in sepsis and acute-on-chronic liver failure (ACLF). Schematic representation of neutrophil immunometabolic states in sepsis and ACLF, highlighting both shared inflammatory programs and disease-specific trajectories. In sepsis (top panel), neutrophils transition from acute inflammatory activation characterized by increased glycolysis, pentose phosphate pathway (PPP) activity, reactive oxygen species (ROS) generation, and NETosis, toward a phase of metabolic conflict involving mitochondrial dysfunction, AMPK–mTOR imbalance, ATP depletion, and eventual immunoparalysis marked by impaired phagocytosis, inhibitory signaling, and functional exhaustion. In ACLF (bottom panel), neutrophils arise from a background of chronic liver disease-associated immune and metabolic priming characterized by persistent endotoxemia, low-grade inflammation, altered lipid metabolism, and basal oxidative stress. Acute decompensation drives exaggerated inflammatory activation alongside early antimicrobial dysfunction, metabolic inflexibility, persistent NETosis, and progressive immune exhaustion. The figure emphasizes a key conceptual distinction between the syndromes: sepsis reflects an acute activation-to-exhaustion trajectory in a previously homeostatic immune system, whereas ACLF develops within a chronically primed and metabolically constrained inflammatory environment. These states are conceptual and likely represent heterogeneous and overlapping neutrophil populations rather than strictly sequential phases. Abbreviations: ACLF, acute-on-chronic liver failure; AMPK, AMP-activated protein kinase; ATP, adenosine triphosphate; CLD, chronic liver disease; DAMPs, damage-associated molecular patterns; mTOR, mechanistic target of rapamycin; NETosis, neutrophil extracellular trap formation; OXPHOS, oxidative phosphorylation; PAMPs, pathogen-associated molecular patterns; PPP, pentose phosphate pathway; ROS, reactive oxygen species.

In sepsis, neutrophil dysfunction reflects an acute transition from hyperactivation to progressive immune paralysis (**Figure 1**). In severe sepsis, neutrophil dysregulation manifests as both hyperactivation and immune paralysis (6). Early in sepsis, neutrophils are hyper-inflammatory; overproduction of ROS and NETs attempts to control pathogens, but systemic NETosis in the circulation drives microvascular occlusion and endothelial injury (30, 31). Critically, pathogen signals and DAMPs upregulate neutrophil survival (e.g. MCL-1 expression) and activate Toll-like receptors, yet simultaneously induce desensitization of chemokine receptors, resulting in “left-behind” neutrophils that fail to migrate to infection sites due to defective CXCR2-dependent chemotaxis and are instead aberrantly recruited (CCR2-mediated) to distant organs, where they release cytotoxic contents and cause tissue injury (32). This impaired chemotaxis is one of the most prominent features of neutrophil dysfunction in sepsis. Normally, neutrophils migrate rapidly to sites of infection under the guidance of chemokines such as IL-8; however, in sepsis, persistent exposure to microbial products such as lipopolysaccharide (LPS) leads to downregulation of chemokine receptors including CXCR2, rendering neutrophils less responsive to chemotactic signals (30, 33, 34). This results in ineffective pathogen clearance and contributes to the uncontrolled spread of infection (35). Hypoxia and metabolic stress in the septic microenvironment further compromise neutrophil motility and effector functions (12).

Neutrophil phagocytic capacity is also severely impaired in sepsis. Neutrophils from septic patients exhibit reduced ability to engulf and kill bacteria, a defect linked to mitochondrial dysfunction, diminished ATP production, and excessive ROS generation during the hyperactivation phase (10, 36). While ROS are essential for microbial killing, their overproduction damages host tissues and promotes a state of exhaustion, rendering neutrophils progressively less effective at pathogen clearance (37). This exhaustion is further exacerbated by continuous recruitment of immature neutrophils from the bone marrow through emergency granulopoiesis, which dilutes the pool of functionally competent neutrophils (38).

Neutrophil extracellular traps or NET formation is another key feature of neutrophil dysfunction in sepsis. NETs are mesh-like structures composed of DNA, histones, and antimicrobial proteins released to trap and kill pathogens; however, excessive release of NETs lead to collateral tissue damage, microvascular thrombosis, and multi-organ failure (39). NETs have been implicated in cardiac, hepatic, and pulmonary dysfunction, and circulating NET markers correlate with disease severity and mortality (40–43). Recent studies have also highlighted there maybe interplay between NETs and ferroptosis, an iron-dependent form of cell death that further exacerbates organ injury in sepsis (44).

In later stages of sepsis, neutrophils may shift toward an immunosuppressive phenotype. Expression of immune checkpoint molecules such as PD-L1 suppresses T-cell responses and contributes to immunoparalysis. Expansion of PD-L1^+^ and other immunosuppressive neutrophil subsets is associated with increased susceptibility to secondary infections and poor clinical outcomes (45, 46). Together, these features define sepsis as a model of acute neutrophil activation followed by metabolic and functional exhaustion.

### 2.2 Neutrophil dysfunction in ACLF

In contrast to sepsis, neutrophil dysfunction in ACLF arises within a chronically primed and metabolically constrained immune environment. ACLF develops due to an acute insult over underlying chronic liver disease, characterized by a biphasic immunopathology characterized by an initial systemic inflammatory response followed by immunosuppression, similar to sepsis (3, 47, 48). This co-existence of immunoparesis with inflammation is now recognised as a hallmark of ACLF (49).

In ACLF patients, neutrophil counts are often elevated with enhanced expression of adhesion molecules such as CD177 however, ACLF neutrophils exhibit marked functional impairments such as reduced phagocytic capacity and bacterial killing suggesting that they drive tissue inflammation while incapable of pathogen elimination (16, 50) These neutrophils display abnormally high resting ROS levels and spontaneous NET formation, along with dysregulated pattern-recognition receptor signaling and defective degranulation of key antimicrobial enzymes (51). In acute alcoholic hepatitis, increased expression of immune checkpoint molecules such as PD-1 and TIM-3, is associated with impaired innate and adaptive responses (52). Neutrophils from patients with HBV-ACLF exhibit impaired phagocytosis, increased tissue migration, reduced TLR expression, impaired bacterial clearance and increased spontaneous NETosis which can be attenuated by the inhibition of glycolysis with 2-deoxyglucose (14, 53).

Neutrophils in ACLF exhibit increased CXCR1/2 expression, which promotes excessive hepatic recruitment and direct neutrophil-hepatocyte interactions that induce apoptosis and necrosis (54). Pharmacological inhibitions of this axis reduces hepatocyte death and dampens pro-inflammatory cytokine and chemokine production, highlighting its therapeutic potential (54). Clinically, elevated absolute neutrophil counts and functional defects including impaired phagocytosis, dysregulated ROS production and reduced complement expression, are strongly associated with higher mortality (16, 48). In contrast, in sepsis, chemokine receptor desensitization and aberrant tissue sequestration emerge secondary to metabolic exhaustion, reflecting failure to sustain coordinated migratory and effector programs (6, 33).

Thus, while both syndromes exhibit neutrophil-driven inflammation with impaired antimicrobial function, the underlying drivers differ fundamentally. These shared and disease-specific features are summarized in **Box 1**.

## 3 Metabolic reprogramming of neutrophils in inflammation

We next examine how metabolic pathways are similarly engaged yet differentially regulated in ACLF and sepsis, shaping neutrophil function across these syndromes. Neutrophil dysfunction in critical illness reflects metabolic changes shaped by the disease context, rather than isolated signaling defects. In inflammatory environments characterized by hypoxia, nutrient limitation, and sustained cytokine exposure, neutrophils must continuously adapt their metabolic circuitry to meet energy and redox demands (55). While traditionally viewed as predominantly glycolytic cells, they are now known to engage a wider metabolic network which include mitochondrial respiration, fatty acid oxidation, amino acid metabolism, and the pentose phosphate pathway, depending on the activation state and tissue microenvironment (11, 56). These adaptations sustain inflammatory output but, when prolonged, compromise antimicrobial function and promote immune exhaustion (6, 57).

### 3.1 Disease-specific metabolic trajectories of neutrophils in ACLF and sepsis

**Figure 2:**
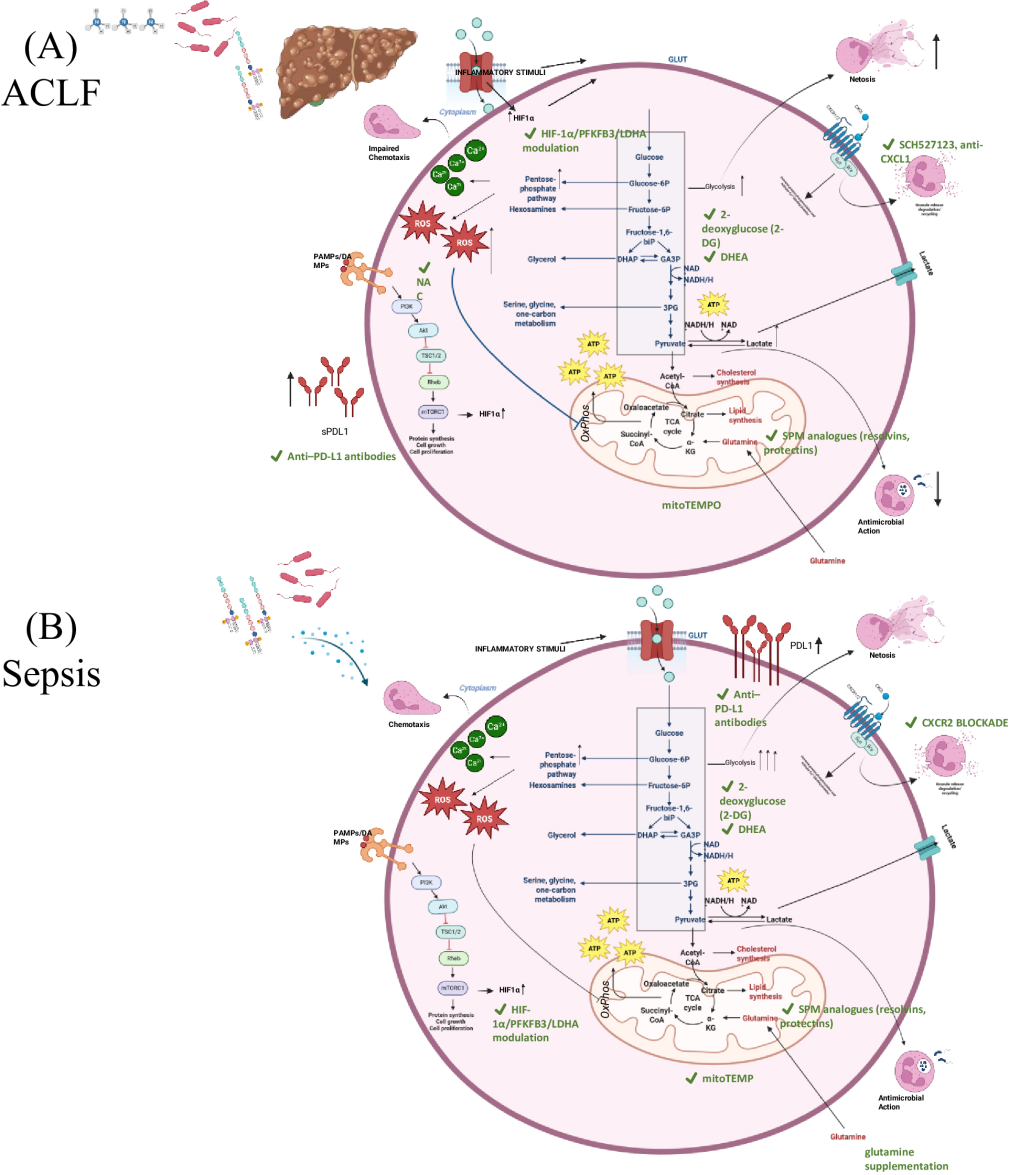
Metabolic reprogramming of neutrophils in ACLF and sepsis, and therapeutic strategies aimed at correcting maladaptive immune metabolism. In both ACLF and sepsis, danger signals and pathogen-associated molecular patterns (PAMPs) activate toll-like receptors (TLRs), initiating phosphoinositide 3-kinase (PI3K)-Akt-mechanistic target of rapamycin (mTOR)-hypoxia inducible factor-1α (HIF-1α) signalling. This enhances glucose uptake and drives glycolysis via 6-phosphofructo-2-kinase/fructose-2,6-bisphosphatase 3 (PFKFB3) and lactate dehydrogenase A (LDHA), increasing adenosine triphosphate (ATP) and nicotinamide adenine dinucleotide phosphate (NADPH) production through the pentose phosphate pathway (PPP), thereby promoting nicotinamide adenine dinucleotide phosphate oxidase 2 (NOX2)-dependent reactive oxygen species (ROS) generation and neutrophil extracellular trap (NET) formation. As glycolysis dominates, oxidative phosphorylation (OXPHOS) activity falls, the tricarboxylic acid (TCA) cycle slows, lactate accumulates, and redox balance is disturbed, reshaping neutrophil functions including chemotaxis, phagocytosis, NETosis, and microbial killing. **(A)** ACLF, neutrophils are pre-activated even before acute insult due to chronic exposure to bacterial products, bile acids, and ammonia, resulting in impaired chemotaxis, high ROS, and early mitochondrial injury. Therapies here target liver-primed dysfunction: C-X-C motif chemokine receptor 1/2 (CXCR1/2) blockade (SCH527123) and anti-CXCL1 limit harmful hepatic recruitment; N-acetylcysteine (NAC) restores glutathione and redox tone; 2-deoxyglucose (2-DG) and HIF-1α/PFKFB3/LDHA modulation reduce early hyper-glycolysis; mitoTEMPO protects mitochondria; specialized pro-resolving mediators (SPMs; resolvins/protectins) support inflammatory resolution; and anti-programmed death ligand-1 (PD-L1) antibodies are proposed to reverse exhaustion. **(B)** sepsis, neutrophils mount an acute glycolytic surge with high ROS and NETosis early, followed by metabolic stress, impaired OXPHOS, lactate accumulation, and functional paralysis despite ongoing inflammation. Interventions therefore align with disease stage: CXCR2 antagonists may restore migration after receptor desensitisation; glutamine supplementation supports oxidative burst during later phases; glycolytic/HIF-1α axis inhibition controls early inflammation; and mitoTEMPO, SPM analogues, and anti-programmed death-1 (PD-1)/PD-L1 therapies aim to reduce mitochondrial stress, enhance inflammatory resolution, and reverse checkpoint-mediated immune suppression.

While sepsis and ACLF share core inflammatory features, neutrophil dysfunction in these syndromes arises from distinct metabolic starting states and trajectories (**Box 1**; **Figure 2**).

Box 1 **Similarities and contrasts in neutrophil dysfunction between sepsis and ACLF.**

Shared core features

(1) Sustained PAMP/DAMP exposure prolongs neutrophil survival and suppresses apoptosis.

(2) Metabolically supported inflammatory programs drive ROS generation and NET formation.

(3) Dysregulated neutrophil death pathways amplify tissue injury while impairing bactericidal capacity.

(4) Both syndromes converge on inflammation with declining antimicrobial efficiency.

Key contrasts

(1) Baseline state:

 *Sepsis* - acute activation in a previously homeostatic immune system.

 *ACLF* - collapse of a chronically primed, metabolically stressed neutrophil system.

(2) Trafficking defects:

 *Sepsis* - CXCR2 desensitization leads to failed migration to infection sites and aberrant organ sequestration.

 *ACLF* - enhanced CXCR1/2 expression promotes excessive hepatic recruitment and local tissue injury.

(3) Metabolic trajectory:

 *Sepsis* - early hyperactivation followed by mitochondrial dysfunction and exhaustion.

 *ACLF* - sustained glycolytic activation with high basal oxidative stress and early loss of antimicrobial competence.

(4) Phagocytosis:

 *Sepsis* - progressively impaired due to energetic failure and dilution by immature neutrophils.

 *ACLF* - intrinsically defective, reflecting dysregulated pattern-recognition and granule release.

(5) NETosis:

 *Sepsis* - excessive, systemic NET release contributes to microvascular thrombosis.

 *ACLF* - spontaneous NET formation reflects chronic pre-activation.

(6) Immunosuppressive shift:

 *Sepsis* - late acquisition of checkpoint-mediated immunosuppression (e.g., PD-L1).

 *ACLF* - functional suppression despite ongoing inflammatory recruitment.

Framework insight: Sepsis follows an acute activation to exhaustion trajectory, whereas ACLF reflects failure of a chronically primed neutrophil state, leading to disease- and stage-specific immunometabolic interventions.

In sepsis, neutrophil metabolic reprogramming is triggered by an acute systemic inflammatory insult in a previously homeostatic immune environment. Early disease is characterized by a rapid shift toward glycolysis, supporting acute effector activation and inflammatory output (58, 59). However, sustained inflammatory signalling rapidly exceeds mitochondrial adaptive capacity, leading to impaired oxidative phosphorylation, ATP depletion, and accumulation of metabolic by-products such as lactate (36, 60). As a result, neutrophils transition from early hyperactivation to metabolic exhaustion, with progressive loss of antimicrobial efficiency and coordinated migration (6, 61).

In contrast, neutrophil metabolism in ACLF is shaped by chronic, pre-existing metabolic stress imposed by liver dysfunction. Persistent endotoxemia, altered lipid handling, and accumulation of toxic metabolites drive sustained glycolytic reliance and elevated basal oxidative stress even prior to acute decompensation (13, 14, 36). Rather than mounting an effective response, neutrophils operate within a metabolically constrained state in which inflammatory activity persists despite impaired antimicrobial competence (14, 39). This has been shown mainly in context of HBV-associated ACLF. However, ACLF arises from diverse etiologies, including alcohol-associated liver disease and metabolic dysfunction-associated steatotic liver disease (MASLD/MASH), and the nature of chronic immune and metabolic priming is likely to differ across these conditions (62). The nature of immune and metabolic priming likely differs across these conditions. However, comparative immunometabolic data across ACLF etiologies remain limited, representing an important area for future investigation.

These distinct metabolic origins shape downstream neutrophil behaviour (**Figure 2**).

Thus, sepsis follows an acute glycolytic activation-to- mitochondrial exhaustion trajectory, whereas ACLF reflects dysfunction within a chronically primed and metabolically constrained immune state. Recognizing these differences is essential for developing disease- and stage-specific immunometabolic therapies (**Box 1**).

## 4 Immunometabolic states of neutrophils: from hyperactivation to immunoparalysis

In this section, we explore the temporal progression of neutrophil immunometabolic states from activation to exhaustion.

Neutrophils, rapidly adapt their metabolism to the shifting demands of inflammation and tissue injury. They are thought to transition across a spectrum of immunometabolic states, ranging from early hyperactivation to later functional exhaustion. While this conceptual trajectory is well supported in sepsis (6) and partially inferred in ACLF (18), it is not clear whether it exists as a unified continuum in either condition.

These trajectories are not identical across syndromes: sepsis reflects an acute transition, whereas ACLF evolves on a background of chronic immune and metabolic priming. These conceptual states are illustrated in **Figure 1**, which integrates metabolic programs with neutrophil maturation and functional outputs, including ROS production, NET formation, and phagocytic capacity. These depicted states are conceptual and represent overlapping rather than strictly sequential states, and are influenced by neutrophil maturation, with immature or low-density subsets exhibiting altered metabolism and impaired antimicrobial function.

### 4.1 Hyperactivation

The Acute Phase: In the hyperactivation phase, the initial cytokine storm which is characterized by high levels of IL−6, IL−8, TNFα, and colony−stimulating factors that drives neutrophils into a hypermetabolic state marked by upregulated glycolysis and pentose−phosphate pathway (PPP) flux (58). In sepsis enhanced glucose uptake and PPP activity, generate NADPH for an explosive respiratory burst and NETosis. PPP activity, regulated in part by glucose-6-phosphate dehydrogenase, sustains NADPH oxidase–dependent ROS production, linking metabolic flux to antimicrobial function. NET formation is further regulated by PAD4-mediated chromatin decondensation (63).

In ACLF, neutrophils exhibit increased glycolytic activity and spontaneous NET formation, alongside elevated circulating histone–DNA complexes (14, 64, 65).

Complement signalling, via C5a prolongs neutrophil survival and amplifies NOX2−dependent ROS, and C5aR1 inhibition reduces ROS (66, 67). Rapid degranulation follows, releasing myeloperoxidase (MPO) and elastase; which correlate with severity in sepsis, and ACLF (68, 69). Although primarily fueled by glycolysis, neutrophils also engage limited glutamine and fatty acid oxidation to sustain chemotaxis, phagocytosis, and degranulation. This metabolic plasticity is critical for immediate host defense but prone to rapid energy depletion and collateral damage if prolonged (55, 70).

### 4.2 The transition phase

As inflammation persists, neutrophils enter a phase of metabolic stress and regulatory checkpoint activation. Nutrient deprivation and hypoxia intensify competition for glucose and oxygen, engaging sensors such as mTOR and AMPK (8, 71). Excessive ROS and accumulated TCA intermediates activate AMPK to conserve ATP, while mTORC1 suppression under nutrient stress shifts neutrophils toward catabolic processes like autophagy. In septic mice, AMPKα1 deficiency prevents this metabolic downshift, leading to relentless ROS production and accelerated exhaustion (72). Concurrently, inhibitory receptors are upregulated: PD−L1 expression increases on septic neutrophils via STAT3, and its blockade restores chemotaxis and phagocytosis. In ACLF-related conditions, similar checkpoint pathways are engaged; increased PD-1 and TIM-3 signaling has been shown to suppress immune function, while their blockade restores T-cell cytokine production and improves neutrophil antimicrobial responses, indicating that immunoparalysis is an actively regulated and potentially reversible state (52, 73, 74). Chronic TLR4 stimulation induces GRK2, internalizing CXCR2 and impairing directed migration, an effect mirrored in cirrhotic endotoxemia (33, 71).

This phase reflects a balance between sustained activation and metabolic stress, with checkpoint pathways limiting excessive inflammation at the cost of antimicrobial efficiency.

### 4.3 Immunoparalysis

**Figure 3:**
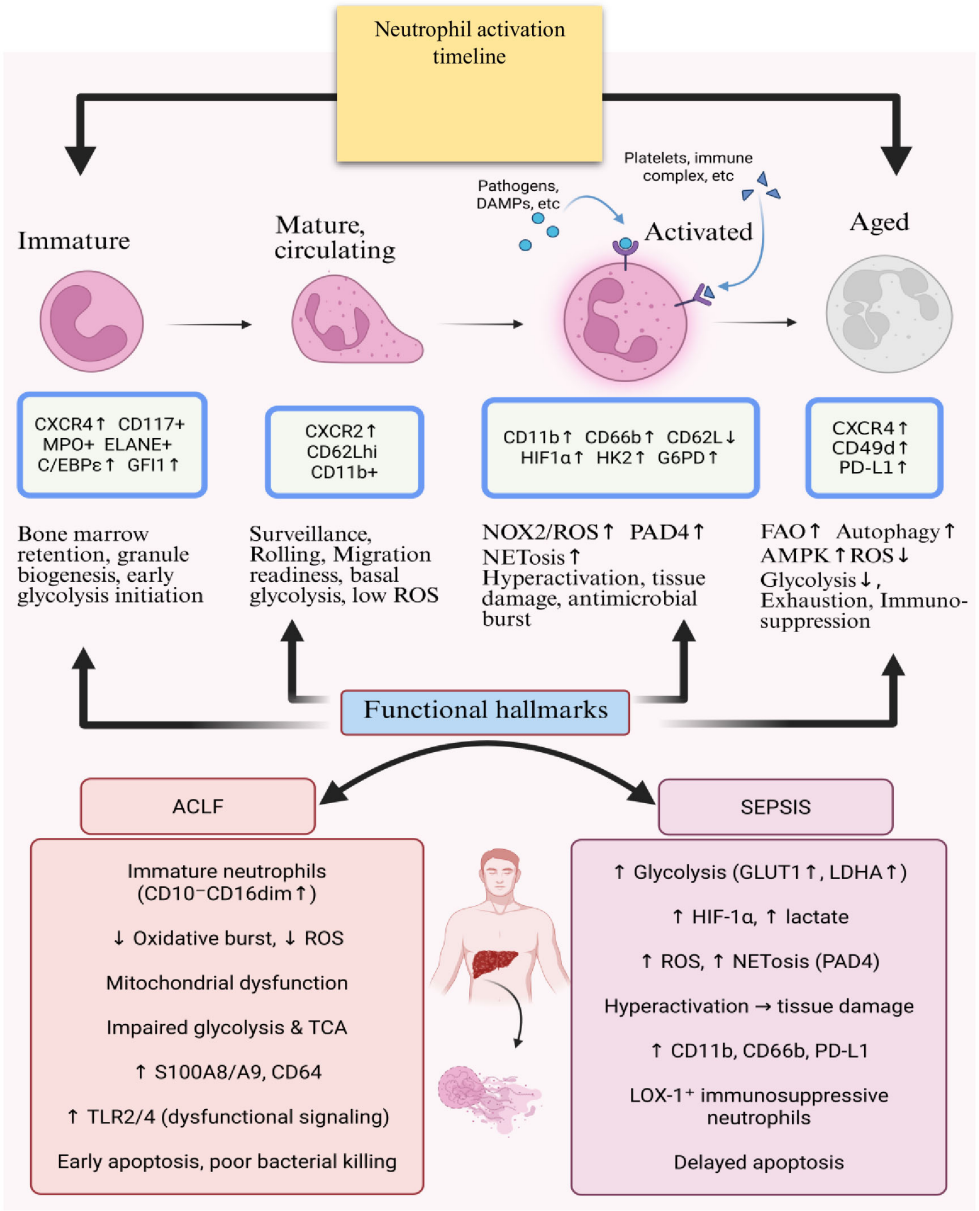
Trajectory of neutrophil maturation with disease-specific immunometabolic signatures in sepsis and ACLF. Trajectory of neutrophil maturation with disease-specific immunometabolic signatures in sepsis and ACLF. The schematic illustrates the continuous progression from early immature to terminally activated neutrophil states, annotated with key phenotypic and metabolic markers. Right panel compares neutrophils from patients with sepsis and ACLF, highlighting distinct immunometabolic reprogramming: sepsis neutrophils exhibit enhanced glycolysis, ROS production, NETosis and delayed apoptosis, whereas ACLF neutrophils display impaired oxidative burst, mitochondrial dysfunction, increased immature subsets, and reduced antimicrobial activity. The figure summarizes how different clinical contexts drive divergent neutrophil functional states.

If inflammation persists, neutrophils progress to immunoparalysis, characterized by metabolic exhaustion and functional decline (6). Downregulation of glycolytic enzymes (hexokinase 2, PFK1) leads to critical ATP depletion and impaired oxidative burst thereby compromising immune effector functions. Mitochondrial membrane potential dissipates, cytochrome c is released, and apoptotic pathways become predominant (6). Phagocytic capacity declines in both sepsis and ACLF; however, while mechanistically defined in sepsis, evidence in ACLF is largely based on functional assays such as bacterial uptake and linked to clinical outcomes (48). Expansion of immunosuppressive granulocytic myeloid-derived suppressor cells further inhibits immune responses through arginine depletion and T-cell suppression (75). Increased proportions of immature neutrophils contribute to altered metabolic capacity and impaired host defense. This terminal state is marked by depleted metabolic reserves, ineffective antimicrobial function, and increased susceptibility to secondary infections and organ failure. Expansion of immature neutrophil populations further contributes to immunoparalysis and altered metabolic capacity, consistent with the maturation-associated shifts (**Figure 3**). The key principles underlying these transitions are summarized in **Box 2**, which integrates stage-specific metabolic states with disease context and therapeutic implications.

Box 2 **An Immunometabolic Framework for Neutrophil Dysfunction in ACLF and Sepsis.**

1. Neutrophil dysfunction in critical illness reflects dynamic metabolic states, not binary activation or suppression.
2. Glycolytic dominance sustains early inflammatory effector functions but predisposes to oxidative stress and exhaustion.
3. Disease context matters: ACLF neutrophils are chronically primed by hepatic metabolic stress, whereas sepsis neutrophils undergo abrupt activation followed by collapse.
4. Mitochondrial inflexibility marks the transition from hyperactivation to immunoparalysis limiting energy adaptation and antimicrobial function.

Therapeutic vulnerability is stage-dependent, requiring temporal and tissue-specific immunometabolic

Targeting key “metabolic checkpoints”, including pathways involving PFKFB3, AMPK–mTOR signaling, and immune checkpoint regulation may therefore offer opportunities to modulate neutrophil responses in ACLF, sepsis, and other inflammatory conditions. Given the current reliance on cross-sectional and heterogeneous datasets, this trajectory should be interpreted as a conceptual framework requiring further validation through longitudinal and mechanistic studies. Emerging evidence suggests that cellular stress-response pathways, particularly endoplasmic reticulum (ER) stress, contribute to immunometabolic regulation (76–78). The PERK pathway links metabolic stress to inflammatory signaling through ATF4, CHOP, and NF-κB, influencing cytokine production and cell fate (79, 80). Sustained activation may promote apoptosis and tissue injury. TRIM29 has been identified as a regulator of innate immune responses and may modulate PERK-dependent ER stress signaling. Although its role in neutrophils remains unclear, the TRIM29–PERK axis may represent an additional layer of immunometabolic control as ER stress and UPR activation have been implicated in a range of inflammatory conditions, including sepsis (81–84). Further investigation is needed, particularly in ACLF, where chronic metabolic stress may amplify ER stress responses.

### 4.4 Epigenetic and long-term reprogramming of neutrophil function

Beyond acute metabolic adaptation, neutrophil dysfunction may also reflect longer-term epigenetic and metabolic reprogramming. Under sustained inflammatory stress, particularly hypoxia, neutrophil progenitors can undergo durable changes that persist beyond the initial insult. Hypoxia-induced chromatin remodeling, including loss of activating histone marks such as H3K4me3 and histone clipping, has been shown to impair antimicrobial function over time (85). In sepsis, such mechanisms may contribute to persistent immune dysfunction even after clinical recovery, consistent with prolonged immunosuppression following acute inflammatory stress (86). In contrast, ACLF represents a state of chronic metabolic and inflammatory priming, where repeated insults may reinforce maladaptive programming. Although direct evidence in ACLF remains limited, these observations suggest that neutrophil dysfunction may not be purely transient but may instead reflect sustained or imprinted immune dysregulation. Understanding these longer-term regulatory mechanisms is critical for distinguishing reversible dysfunction from persistent immune impairment in critical illness.

## 5 Therapeutic reprogramming: reversing immunometabolic fate

The shared and disease-specific immunometabolic mechanisms discussed above provide a conceptual basis for therapeutic reprogramming. Given their central role in both antimicrobial defense and tissue injury, neutrophils represent attractive targets for therapeutic reprogramming. However, many of these strategies are not neutrophil-specific and may exert broader effects on hepatic function, systemic metabolism, tissue repair, and host defense. Therefore, current therapeutic strategies aim to target key metabolic and inflammatory checkpoints that regulate neutrophil activation, trafficking, and immunosuppressive signaling. Insights from sepsis provide proof-of-concept, but ACLF presents unique challenges due to its liver-centric pathology and the chronic immune priming of cirrhosis. Accordingly, several immunometabolic pathways have emerged as potential therapeutic targets, although most remain supported primarily by mechanistic and preclinical evidence.

### 5.1 Modulation of chemokine receptor signaling

The CXCL1-CXCR1/2 axis is a key determinant of hepatic neutrophil infiltration and hepatocyte injury in ACLF. Inhibition of CXCR1/2 with small-molecule antagonists such as SCH527123 reduces neutrophil infiltration and parenchymal death in preclinical ACLF models (87). Similarly, CXCL1 blockade diminishes neutrophil recruitment and oxidative stress (88). These findings highlight liver-targeted chemokine receptor antagonism as a strategy to selectively prevent tissue damage. In sepsis, CXCR2 blockade has also been tested to restore neutrophil trafficking, but requires precise timing to avoid impairing host defense (32, 89).

One aspect we must take cognizance of, however, is that chemokine receptor signaling does not operate in isolation but is highly sensitive to the metabolic state of neutrophils (90). In disease, systemic metabolic disturbances can disrupt chemokine-guided migration even in the presence of intact receptor expression. In sepsis, extracellular ATP accumulates in the circulation as a consequence of widespread tissue injury and cellular stress (91). Elevated plasma ATP perturbs purinergic signaling, impairing neutrophil polarization and directional sensing, thereby inhibiting effective chemotaxis. In an *E. coli* peritonitis model, enzymatic reduction of systemic ATP levels using apyrase restores neutrophil chemotaxis and reduces pathogen burden, providing direct evidence that metabolic dysregulation upstream of chemokine signaling contributes to migratory failure in sepsis (92).

Beyond ATP availability, intracellular metabolic stress responses further constrain chemotactic signaling. Disruption of glucose recycling from the endoplasmic reticulum induces ER stress, elevates Hsp90 and cellular reactive oxygen species, and activates HIF-1α dependent transcriptional programs (93). Subsequent activation of PPAR-γ inhibits ERK-1/2 MAPK signalling downstream of chemotactic stimuli, thereby suppressing the intracellular signalling cascades required for directed migration (94). These observations indicate that metabolic stress can impair neutrophil chemotaxis not only by altering chemokine receptor expression or sensitivity, but also by rewiring intracellular signalling pathways essential for migratory competence.

Together, these findings position metabolic reprogramming as an upstream regulator of CXCR1/2-dependent neutrophil trafficking in critical illness. In both sepsis and ACLF, metabolic stress manifesting as ATP accumulation, ER stress, and glycolytic dominance, creates a cellular environment in which chemotactic cues are present but cannot be effectively transduced into coordinated migration, contributing to immune inefficiency and organ injury.

### 5.2 Regulation of glycolytic flux and HIF-1α pathways

Excessive glycolytic flux serves as a central metabolic driver of neutrophil hyperactivation, sustaining NETosis and amplifying pro-inflammatory effector functions in both syndromes. This hyperglycolytic state is transcriptionally reinforced through the HIF-1α - PFKFB3- LDHA axis, which collectively accelerates glycolytic throughput, enforces lactate production, and maintains redox balance required for sustained inflammatory output (95). Pharmacologic inhibition of glycolysis using 2-deoxy-glucose (2-DG), as well as targeted modulation of key nodes within this axis (such as LDHA), has been shown to attenuate neutrophil activation, NET formation, and tissue-damaging inflammatory responses (96, 97). However, emerging evidence indicates that late-stage immunoparalysis is itself associated with metabolic insufficiency and impaired glycolytic capacity (74, 98). Consequently, metabolic interventions targeting glycolysis must be applied in a temporally and context-specific manner so as to restrain early hyperinflammation while preserving, or even restoring, metabolic competence during later phases to avoid exacerbating immune exhaustion and susceptibility to secondary infections.

### 5.3 Restoration of mitochondrial fitness and redox balance

Neutrophil mitochondrial dysfunction has emerged as a critical determinant of impaired phagocytic capacity and aberrant survival in severe inflammatory states. Although neutrophils rely predominantly on glycolysis, intact mitochondrial function is essential for regulating apoptosis, calcium handling, and the controlled generation of mitochondrial reactive oxygen species (mtROS), which function as signaling mediators rather than purely cytotoxic by-products (99, 100) Mitochondrial depolarization and excessive mtROS disrupt cytoskeletal dynamics and phagosome maturation, leading to defective bacterial clearance while simultaneously delaying apoptotic turnover and promoting the persistence of dysfunctional neutrophils (101–104). Accordingly, therapeutic strategies aimed at restoring mitochondrial homeostasis, including supplementation with metabolic substrates, selective scavenging of mtROS using mitochondria-targeted antioxidants such as mitoTEMPO, and replenishment of intracellular antioxidant capacity with agents such as N-acetylcysteine, have been shown to improve neutrophil function and facilitate resolution of inflammation (105–107). In ACLF, this mitochondrial vulnerability is further amplified by hyperammonemia-induced mitochondrial stress and profound glutathione depletion, which together exacerbate redox imbalance and neutrophil dysfunction (108–110). In this context, therapeutic approaches that restore redox buffering and mitochondrial signaling fidelity, through mitochondria-targeted interventions, regulation of mitochondrial quality control pathways such as mitophagy and biogenesis, and targeted modulation of metabolic stress-response pathways, may be particularly well suited to correct innate immune failure without exacerbating inflammatory injury (111, 112).

### 5.4 Lipid mediators and specialized pro-resolving mediators

Neutrophil lipid metabolism plays a central role in the initiation of inflammation and also in its active resolution (112, 113). Beyond serving as structural components or energy substrates, lipids function as signaling molecules that shape neutrophil activation thresholds, trafficking, lifespan, and clearance (114). In inflammatory settings, arachidonic acid-derived eicosanoids initially promote neutrophil recruitment and activation; however, effective resolution requires a coordinated lipid mediator class switch toward specialized pro-resolving mediators (SPMs), including resolvins, protectins, maresins, and lipoxins (115, 116).

SPMs exert pleiotropic effects on neutrophils through engagement of specific G-protein coupled receptors, leading to suppression of excessive chemotaxis, limitation of NET formation, and restoration of effective phagocytic and efferocytic capacity (36). Mechanistically, SPM signaling dampens NF-κB and MAPK activation, restrains ROS overproduction, and promotes cytoskeletal reorganization required for bacterial clearance and apoptotic cell uptake, while simultaneously accelerating neutrophil apoptosis and clearance by macrophages (117). Importantly, these actions resolve inflammation without inducing broad immunosuppression, distinguishing SPMs from conventional anti-inflammatory agents.

In sepsis, deficiencies in endogenous SPM production and impaired lipid mediator class switching have been documented, contributing to persistent neutrophil activation, delayed resolution, and ongoing tissue injury (118, 119). Experimental models demonstrate that supplementation with SPMs or stable SPM analogues restores neutrophil phagocytic competence, reduces NET-mediated collateral damage, and enhances bacterial clearance while limiting inflammatory amplification (120). These effects are mediated, in part, by reprogramming neutrophil metabolic and redox states toward resolution-compatible phenotypes.

In ACLF, the relevance of SPM biology may be further amplified. Hepatic dysfunction profoundly disrupts lipid synthesis, remodeling, and clearance, resulting in altered availability of polyunsaturated fatty acid precursors and impaired generation of pro-resolving lipid mediators (121). In this context, defective SPM signaling may contribute to sustained neutrophil activation, impaired efferocytosis, and failure of inflammatory resolution (122). ACLF inflammatory mileu is further worsened by the depletion of circulating lysophosphatidylcholines (LPC) (120, 123, 124). Although direct interventional data in ACLF remain limited, the combination of neutrophil-driven inflammation and disordered lipid metabolism provides a strong mechanistic rationale for exploring SPM-based or lipid-resolution-targeted therapies as adjunctive strategies to restore immune balance without exacerbating inflammatory injury.

### 5.5 Amino acid and immune checkpoint pathways

Amino acid metabolism represents an additional, and often underappreciated, layer of immunometabolic regulation with direct relevance to innate immune dysfunction. For instance, glutamine supplementation has been shown to restore neutrophil oxidative burst capacity in sepsis, highlighting the sensitivity of innate immune effector functions to substrate availability (125). ACLF is similarly characterized by profound perturbations in systemic amino acid homeostasis, with arginine metabolism emerging as a key node linking hepatic injury to extrahepatic complications, particularly blood brain barrier (BBB) dysfunction (126).

In HBV-associated ACLF, circulating arginine levels have paradoxically been reported to be elevated, likely reflecting increased whole-body protein catabolism. However, this occurs in the context of a functionally impaired urea cycle, marked by downregulation of arginase-1, resulting in ineffective nitrogen handling and a persistent metabolic crisis rather than true metabolic sufficiency (126, 127). Consistent with this, oral arginine supplementation has been shown to restore effective circulating arginine pools, attenuate BBB disruption, and reduce neurological dysfunction in experimental models of liver failure (128).

Beyond neuroprotection, arginine supplementation in acute liver injury models significantly limits hepatic necrosis, reduces serum transaminases (ALT, AST), and dampens inflammatory cell infiltration within the liver parenchyma (129). From an immunometabolic perspective, arginine availability supports a shift in immune cell metabolism away from a predominantly glycolytic, pro-inflammatory phenotype toward mitochondrial oxidative phosphorylation (OXPHOS), thereby restraining excessive inflammatory activation, while still permitting regulated nitric oxide (NO) production required for vascular homeostasis (126). In parallel, arginine supplementation has also been shown to reduce bacterial translocation to the liver and mesenteric lymph nodes in acute liver injury, further emphasizing its role at the interface of metabolism, barrier integrity, and innate immune defense (126).

Therapeutic reprogramming of neutrophils is governed by three overarching principles: precise alignment of interventions with disease stage, spatial tailoring to the tissues most critically affected, and careful calibration between preservation of antimicrobial competence and limitation of host-damaging inflammation. In sepsis, stage-dependent metabolic modulation is already being actively explored as a means to restore neutrophil function without exacerbating immune paralysis. In contrast, ACLF may be more amenable to tissue-focused strategies, including modulation of chemokine axes such as CXCR1/2 or targeted redox support within specific microenvironments. Collectively, these observations underscore the feasibility of therapeutically redirecting neutrophil immunometabolic fate, positioning such reprogramming as a unifying framework for next-generation interventions in systemic inflammatory disease.

## 6 Tools and technologies to study neutrophil metabolism

Although neutrophils play a central role in inflammation, their metabolic pathways remain relatively less explored due to their short lifespan, limited transcriptional activity, and technical challenges associated with ex vivo manipulation. However, recent advances in metabolic profiling and single-cell technologies have revealed that neutrophils display remarkable metabolic plasticity that supports their diverse roles during acute and chronic inflammatory conditions, including acute-on-chronic liver failure (ACLF) and sepsis (127). Capturing this metabolic diversity requires a multifaceted methodological toolkit capable of interrogating substrate utilization, pathway fluxes, functional metabolic outputs, and spatiotemporal dynamics (128, 129).

Given the complexity and heterogeneity of neutrophil responses in critical illness, no single technique can fully characterize immunometabolic states. Instead, complementary approaches integrating flux-based analyses, single-cell profiling, spatial methods, functional assays, and multi-omics technologies are required to link metabolic activity with immune behavior in clinically relevant contexts (130, 131).

**Figure 4:**
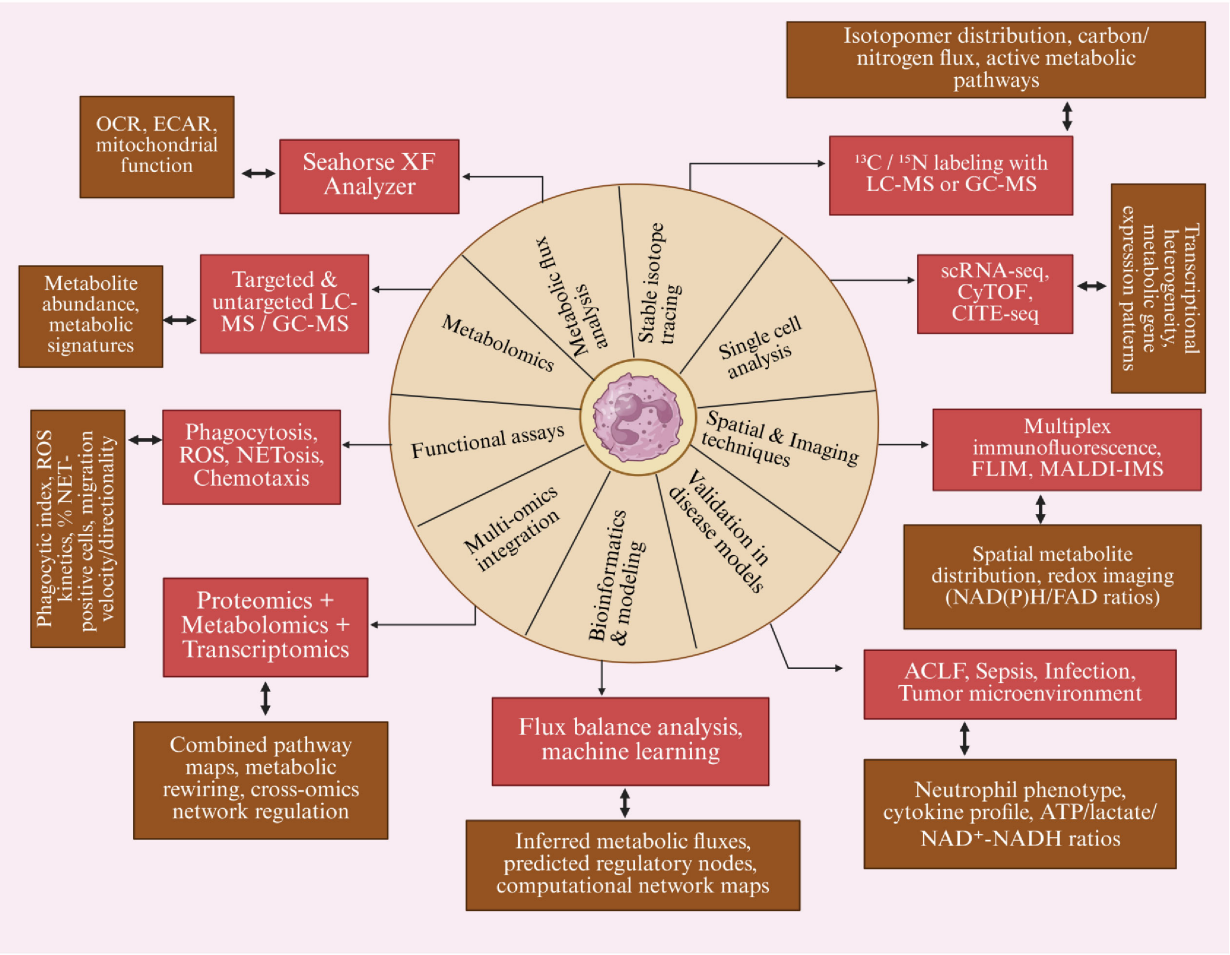
Overview of analytical methods and functional readouts in neutrophil metabolic studies. This figure summarizes the major experimental and computational approaches currently used to investigate neutrophil metabolism and immunometabolic reprogramming. The central framework integrates complementary methodological domains including metabolomics, metabolic flux analysis, stable isotope tracing, single-cell profiling, spatial and imaging approaches, functional assays, multi-omics integration, and computational modelling. Each platform is linked to the principal techniques employed and the biological or metabolic information generated. Extracellular flux analysis using Seahorse XF platforms measures oxygen consumption rate (OCR) and extracellular acidification rate (ECAR) to assess mitochondrial respiration and glycolytic activity. Targeted and untargeted metabolomics performed using liquid chromatography–mass spectrometry (LC-MS) or gas chromatography–mass spectrometry (GC-MS) characterize metabolite abundance and pathway signatures. Stable isotope tracing using ^13C/^15N-labelled substrates enables reconstruction of carbon and nitrogen metabolic fluxes. Single-cell approaches including single-cell RNA sequencing (scRNA-seq), cytometry by time-of-flight (CyTOF), and cellular indexing of transcriptomes and epitopes by sequencing (CITE-seq) reveal transcriptional heterogeneity and metabolic gene-expression programs across neutrophil subsets. Spatial and imaging techniques such as multiplex immunofluorescence, fluorescence lifetime imaging microscopy (FLIM), and matrix-assisted laser desorption/ionization imaging mass spectrometry (MALDI-IMS) enable visualization of metabolic organization and redox states within tissue microenvironments. Functional assays assessing reactive oxygen species (ROS), phagocytosis, neutrophil extracellular trap (NET) formation, chemotaxis, and migratory velocity link metabolic rewiring with immune effector function. Finally, integration of proteomic, metabolomic, and transcriptomic datasets with computational approaches such as flux balance analysis and machine-learning-based network modelling enables inference of metabolic pathway remodeling and regulatory networks across inflammatory conditions including acute-on-chronic liver failure (ACLF) and sepsis.

As illustrated in **Figure 4**, current methodological frameworks for studying neutrophil metabolism include metabolic flux measurements, single-cell profiling, functional assays, and systems-level computational analyses enabling a more comprehensive mapping of immunometabolic states. Extracellular flux analysis using Seahorse XF platforms enables quantification of glycolytic activity and mitochondrial respiration through measurements of extracellular acidification rate (ECAR) and oxygen consumption rate (OCR). In parallel, targeted and untargeted metabolomics using LC–MS or GC–MS characterize metabolite abundance and metabolic pathway signatures, while stable isotope tracing with 13C-glucose or 15N-glutamine labeling enables reconstruction of metabolic fluxes and identification of active carbon and nitrogen pathways. At the cellular level, single-cell technologies such as scRNA-seq, CyTOF, and CITE-seq provide insight into transcriptional heterogeneity and metabolic gene expression programs across neutrophil subsets. Spatial and imaging approaches, including multiplex immunofluorescence, fluorescence lifetime imaging microscopy, and mass spectrometry-based imaging enable the mapping of metabolic states within tissue microenvironments. These molecular measurements can be complemented by functional assays that assess neutrophil effector activities, including reactive oxygen species production, phagocytosis, NET formation, and chemotaxis, thereby linking metabolic rewiring to immune function. Finally, multi-omics integration and computational modeling approaches, such as flux balance analysis and machine-learning based network reconstruction, enable inference of regulatory nodes and metabolic pathway remodeling across inflammatory conditions such as sepsis and ACLF. Detailed descriptions of these methodologies are provided in the Supplementary Methods.

## 7 Critical gaps and conclusion

Despite substantial progress in understanding neutrophil immunometabolism in sepsis and ACLF, several key gaps continue to limit translation into effective therapies. First, the temporal dynamics of neutrophil metabolic reprogramming remain poorly defined, particularly in ACLF. While longitudinal immune profiling has revealed distinct phases of hyperinflammation and immunoparalysis in sepsis (132), most ACLF studies rely on cross-sectional analyses that provide only snapshots of glycolytic skewing or oxidative dysfunction (133). Prospective studies incorporating serial sampling will be necessary to define how neutrophil metabolic states evolve during disease progression and recovery.

Second, spatial context is insufficiently understood. In sepsis, compartmentalized immune phenotypes have been observed across tissues, with neutrophils displaying distinct functional states in organs such as the lung, kidney, and circulation (134–136). Comparable tissue-resolved analyses in ACLF remain limited. The hepatic microenvironment imposes unique metabolic pressures, including hyperammonaemia, altered lipid metabolism, and persistent endotoxemia, yet how these factors shape the behaviour of infiltrating neutrophils relative to circulating populations is largely unknown. Integrated analyses of paired blood and liver samples, together with emerging spatial and imaging approaches, may help clarify these relationships.

Third, the mechanistic regulation of immunometabolic checkpoints remains incompletely defined. Pathways involving glycolysis, mitochondrial reactive oxygen species, and amino-acid metabolism have been implicated in neutrophil dysfunction (56), but the upstream regulatory networks including transcriptional programs, epigenetic modulation, and signaling cascades, are still only partially characterized. A clearer understanding of how these pathways balance antimicrobial defense with inflammatory injury will be essential before metabolic targets can be translated into therapeutic strategies.

Beyond acute metabolic adaptation, neutrophil dysfunction may also be shaped by epigenetic and long-term metabolic reprogramming. Inflammatory and hypoxic stress can induce durable alterations in neutrophil progenitors, including changes in chromatin accessibility and histone modifications, resulting in persistent impairment of antimicrobial function. Hypoxia-driven remodeling of histone marks such as H3K4me3 and histone clipping has been linked to sustained neutrophil dysfunction (85, 137). These mechanisms may be particularly relevant in ACLF, where chronic metabolic and inflammatory stress could promote maladaptive immune imprinting. However, direct evidence in ACLF remains limited and warrants further investigation.

These limitations highlight why precision immunometabolism remains an aspirational goal rather than an immediate clinical reality. Progress will likely depend on integrative approaches that combine longitudinal clinical sampling with single-cell and multi-omics profiling, functional metabolic assays, and computational modelling to map neutrophil states across time and tissue compartments. Such efforts may help shift the field from descriptive profiling toward mechanism-informed interventions. Ultimately, defining when and where neutrophil metabolic programs promote host defense versus tissue injury may allow these pathways to be modulated more selectively in critical illness.
