# Supplementary material for: Neutrophil immunometabolism in ACLF and sepsis: mechanisms, dysfunction, and therapeutic opportunities
Source: Front Immunol. 2026 May 22;17:1828801. doi: 10.3389/fimmu.2026.1828801 (PMC13236496; doi:10.3389/fimmu.2026.1828801)
Supplement: Supplementary file 1 [file DataSheet1.docx]

**5. Tools and Technologies to Study Neutrophil Metabolism**

Although neutrophils play a central role in inflammation, their underlying metabolic pathways and mechanisms are still underexplored due to their short life span, limited transcriptional activity and technical challenges with *ex vivo* manipulation. However, recent advances in metabolic profiling and single cell technologies reveal that neutrophils exhibit remarkable metabolic plasticity that underpins their diverse roles across acute and chronic inflammatory states, including acute-on-chronic liver failure (ACLF) and sepsis (1). Capturing this metabolic diversity requires a multifaceted toolkit capable of interrogating substrate utilization, pathway fluxes, functional metabolic outputs, and spatiotemporal dynamics (2,3).

Given the complexity and heterogeneity of neutrophil responses in critical illness, no single method is sufficient to capture immunometabolic states. Instead, a combinatorial approach integrating flux-based, single-cell, spatial, functional, and multi-omics technologies is required to link metabolism with immune behavior in clinically relevant contexts (4,5).

**5.1. Metabolic Flux Analysis (MFA) as a major analytic tool**

Metabolic flux analysis (MFA) provides quantitative insight into how neutrophils dynamically reroute metabolic pathways during activation, stress, and exhaustion by measuring the flow of substrates through metabolic networks rather than static metabolite abundance.

MFA leverages stable isotopic tracers (e.g., ¹³C-glucose, ¹⁵N-glutamine) to track metabolite fate through pathways. These tracers are introduced into cell cultures, and their incorporation into metabolic intermediates is measured using mass spectrometry (MS) or nuclear magnetic resonance (NMR) spectroscopy. MS provides high sensitivity for detecting isotopic labeling patterns, while NMR offers non-destructive analysis and structural elucidation (6,7). Advanced software tools like INCA (Isotopologue Network Compartmental Analysis), OpenFLUX, and METRAN streamline flux estimation by combining stoichiometric models with isotopic labeling data. These platforms employ algorithms to solve mass balance equations and predict flux distributions across pathways such as glycolysis, the pentose phosphate pathway (PPP), and the tricarboxylic acid (TCA) cycle (6,8).

The MFA workflow begins with experimental design, where tracer selection and labeling time are optimized to capture metabolic steady or non-steady states. An instance is that ¹³C-glucose tracing can elucidate glycolysis and PPP activity, while ¹³C-glutamine reveals TCA cycle dynamics. After quenching metabolic activity, metabolites are extracted and analyzed. Computational models then integrate labeling data with stoichiometric networks to calculate flux rates. Tools like 13CFLUX2 and WUFlux use constraint-based modeling to refine flux predictions, while INST-MFA (Isotopically Non-Stationary MFA) handles dynamic labeling data in non-steady-state conditions (7,9). These models account for reversibility of reactions, compartmentalization, and isotopic scrambling, ensuring accurate flux maps.

In biomedical research, MFA has uncovered metabolic reprogramming in diseases like cancer, where tumor cells exhibit heightened glycolysis (Warburg effect) and glutaminolysis. By quantifying flux through these pathways, MFA identifies therapeutic targets, such as enzymes in nucleotide synthesis or redox balance (10). In autoimmune diseases, MFA reveals how neutrophils shift toward PPP to fuel ROS production and NETosis, exacerbating inflammation (11). In biotechnology, MFA guides metabolic engineering by pinpointing bottlenecks in microbial production systems. For example, flux analysis of *E. coli* or yeast identifies rate-limiting steps in biofuel or pharmaceutical synthesis, enabling targeted overexpression of key enzymes or knockout of competing pathways ([12](https://biotechnologyforbiofuels.biomedcentral.com/articles/10.1186/s13068-019-1372-4)). Again, tools like Flux-P and iMS2Flux automate data processing, enabling high-throughput strain optimization. However, despite its power, MFA faces challenges, including the short half-life of neutrophils and the complexity of eukaryotic metabolism. Single-cell MFA and spatial flux analysis are emerging to address cellular heterogeneity, while multi-omics integration (e.g., transcriptomics + fluxomics) provides a systems-level view ([13](https://doi.org/10.1038/s41580-023-00615-w)). Advances in machine learning further enhance predictive modeling, enabling real-time flux adjustments in bioreactors.

**5.2. Use of single cell and spatial transcriptomics in metabolism research**

Single-cell and spatial technologies have transformed the study of neutrophil immunometabolism by uncovering functional and metabolic heterogeneity that is masked in bulk analyses. Single-cell RNA sequencing (scRNA-seq) identifies transcriptional programs associated with metabolic reprogramming, immature neutrophil subsets, and disease-specific activation states, while spatial transcriptomics preserves tissue context, revealing how local microenvironments shape neutrophil metabolism and function (14-15).

**5.2.1. Met-Flow: Single-Cell Metabolic Profiling by Flow Cytometry**

Met-Flow is a powerful flow cytometry-based technique that enables the simultaneous measurement of metabolic proteins and enzymes at the single-cell level, thus allowing the metabolic state of individual neutrophils to be captured within a heterogeneous population. The core principle of Met-Flow is the use of antibody panels conjugated to fluorochromes that target key metabolic enzymes and proteins involved in various pathways, such as glycolysis, oxidative phosphorylation, and fatty acid metabolism. This approach makes it possible to stain and analyze both metabolic and cell surface proteins in a single assay, providing a high-dimensional view of cell metabolism alongside classical immunophenotyping (15,17) .

The workflow involves isolating neutrophils from blood or tissue, staining them with a panel of antibodies specific to metabolic enzymes and surface markers, followed by high-parameter flow cytometery. Advanced data analysis tools are used to interpret the resulting multidimensional datasets, allowing researchers to identify unique metabolic signatures and correlate them with specific neutrophil subsets or functional states. Applications of Met-Flow include distinguishing metabolic differences between neutrophil subsets, such as CD64+ versus CD64− cells, and linking these profiles to effector functions like cytokine production or NETosis (18,19). The benefits of Met-Flow include its compatibility with standard flow cytometry platforms, the ability to analyze rare cell populations, and the capacity to resolve dynamic metabolic changes at single-cell resolution (16).

**5.2.2. Optical Metabolic Imaging (OMI)**

Optical Metabolic Imaging (OMI) is a label-free, non-destructive technology that uses multiphoton microscopy to measure the intrinsic autofluorescence of metabolic coenzymes NAD(P)H and FAD in live cells. This technique quantifies the optical redox ratio and fluorescence lifetimes, providing real-time insights into the metabolic state of single neutrophils as they respond to stimuli or infection. OMI works by exciting cells with specific wavelengths of light and detecting the emitted fluorescence from NAD(P)H and FAD, which are fundamental to cellular metabolism. The ratio of these signals and their fluorescence lifetimes reflect the redox state of the cell and metabolic activity, allowing researchers to monitor rapid metabolic remodeling in neutrophils (20).

The experimental process involves isolating primary human neutrophils, placing them in appropriate imaging conditions, and then using multiphoton microscopy to collect autofluorescence data under various activation or inhibition scenarios. OMI can be performed both in vitro and in vivo, as demonstrated by studies using zebrafish larvae to validate findings from human cells. The major applications of OMI include tracking metabolic changes during neutrophil activation, oxidative burst, and NETosis, as well as comparing metabolic responses across different donors or disease states (21,22). The advantages of OMI are its non-invasive nature, real-time monitoring capability, and single-cell resolution, making it an ideal tool for studying the immediate and heterogeneous metabolic responses of neutrophils (20,23).

**5.2.3. Seahorse XF analyzer in extracellular flux analysis**

The Seahorse XF Analyzer is a widely used platform for real-time measurement of the oxygen consumption rate (OCR) and extracellular acidification rate (ECAR) in neutrophils, which are indicators of mitochondrial respiration and glycolysis, respectively. This technology is particularly valuable for quantifying the metabolic activity of neutrophil populations in response to various stimuli or treatments. The assay works by plating neutrophils in specialized microplates and using the analyzer sensors to detect changes in oxygen and pH in the extracellular environment as the cells undergo metabolic processes (24).

The workflow involves isolating neutrophils, seeding them onto a Cell-Tak™ coated Seahorse plate, and then sequentially adding mitochondrial inhibitors and activators (such as PMA) to distinguish between mitochondrial and non-mitochondrial oxygen consumption. The analyzer records kinetic traces of oxygen consumption and proton efflux in real time, providing a detailed profile of neutrophil activation and metabolic reprogramming. This method is highly sensitive, non-invasive, and allows for the parallel measurement of glycolysis and oxidative burst, which are critical for understanding neutrophil responses to infection and inflammation. Applications include assessing how neutrophils meet their energetic demands during activation, evaluating the effects of metabolic inhibitors, and studying the requirements for processes like NET formation. The benefits of Seahorse XF technology include its ability to provide real-time, label-free, and kinetic measurements of metabolic activity, which are not possible with traditional endpoint assays (24,25).

**5.2.4. MIBI-TOF (Multiplexed Ion Beam Imaging by Time-of-Flight)**

Multiplexed Ion Beam Imaging by Time-of-Flight (MIBI-TOF) is an advanced spatial proteomics technology that uses metal-tagged antibodies and mass spectrometry to image dozens of proteins simultaneously in tissue sections at subcellular resolution. In MIBI-TOF, antibodies against target proteins—including metabolic enzymes—are conjugated to isotopically pure metal reporters. These labeled antibodies are applied to tissue sections, and the sample is then scanned with a primary ion beam that releases secondary ions from the metal tags. The time-of-flight mass spectrometer detects these ions, allowing for highly multiplexed and spatially resolved protein imaging ([26,27](https://pmc.ncbi.nlm.nih.gov/articles/PMC9636771/)).

The workflow for MIBI-TOF involves preparing tissue sections, performing antibody labeling, and then imaging the samples using the MIBI-TOF instrument. The resulting data can be analyzed to map the expression of metabolic proteins in the context of cell identity, location, and microenvironmental factors. This technology is particularly useful for studying the spatial organization of metabolic heterogeneity within immune cell niches, such as neutrophil-rich regions in inflamed or tumor tissues. MIBI-TOF offers the advantage of high multiplexing capacity (over 40 parameters), subcellular spatial resolution, and compatibility with formalin-fixed, paraffin-embedded (FFPE) tissue, making it suitable for retrospective studies (26,27).

**5.2.5. Spatial Transcriptomics**

Spatial transcriptomics is one of the latest transformative technologies that enables the measurement of gene expression across tissue sections while preserving spatial information. Platforms like 10x Genomics Visium allow researchers to map the localization and metabolic gene expression of neutrophils within complex tissue microenvironments. The method involves placing tissue sections onto slides containing spatially barcoded capture probes, followed by RNA extraction, reverse transcription, and sequencing. The spatial barcodes allow the resulting gene expression data to be mapped back to specific locations within the tissue (15,28).

This workflow enables the integration of spatial transcriptomic data with single-cell RNA-seq, providing a high-resolution view of how spatial context influences neutrophil metabolic states and functions. Applications include studying neutrophil localization and metabolic gene expression in inflamed or tumor tissues, identifying spatially restricted metabolic programs, and understanding cell-cell interactions within the tissue microenvironment. The main benefits of spatial transcriptomics are its ability to provide spatially resolved transcriptomic profiles and its compatibility with other single-cell technologies, offering a comprehensive understanding of tissue organization and function (28, 29).

**5.2.6. Integrative and emerging approaches**

Recent advances have enabled the integration of single-cell metabolic profiling methods, such as Met-Flow and OMI, with spatial technologies like MIBI-TOF and spatial transcriptomics. This multidisciplinary approach allows researchers to correlate metabolic phenotypes with spatial localization and microenvironmental cues, providing unprecedented insight into the complexity of neutrophil immunometabolism in health and disease (24-26,30). Such integrative strategies are essential for understanding the functional specialization and metabolic heterogeneity of neutrophils within tissues. The development of single-cell and spatial technologies has revolutionized the study of neutrophil metabolism. Techniques such as Met-Flow, Optical Metabolic Imaging, Seahorse XF Analyzer, MIBI-TOF, and spatial transcriptomics each offer unique advantages for dissecting the metabolic states and functions of neutrophils. By combining these complementary approaches, researchers can achieve a comprehensive and nuanced understanding of neutrophil immunometabolism, ultimately informing new therapeutic strategies for diseases involving immune dysfunction.

**5.3. Functional and phenotypic assays for neutrophil immunometabolism**

Neutrophil functional assays are essential tools for dissecting the immunometabolic pathways that govern the roles these cells play in immunity and inflammation. This section details key methodologies for assessing reactive oxygen species (ROS) production, phagocytosis, NETosis, chemotaxis, degranulation, and integrated multiplexed screening. Each assay encompasses its working principles, experimental workflow, applications, and benefits, providing a comprehensive resource for investigating neutrophil biology (31).

**5.3.1. Reactive Oxygen Species (ROS) Production Assays**

Reactive oxygen species generation is a critical neutrophil effector function measured to assess metabolic activity during oxidative burst. The chemiluminescence-based assay quantifies ROS by detecting light emission from luminol oxidation during respiratory bursts. Neutrophils are isolated via density-gradient centrifugation or immunomagnetic separation, then stimulated with agents like phorbol myristate acetate (PMA) or TNF-α. ROS production is measured in real-time using plate readers, with immunomagnetic-isolated neutrophils showing higher sensitivity to priming agents like TNF-α and pathogen-associated molecular patterns (e.g., CpG, PGN) (32,33). This assay reveals how purification methods impact metabolic responses, with density-gradient isolates exhibiting suppressed ROS responses post-priming. For high-throughput screening, microfluidic devices enable ROS quantification directly from microliters of whole blood within 10 minutes, eliminating lengthy isolation steps (34). These platforms use fluorescent probes (e.g., dihydrorhodamine 123) oxidized by ROS, detected via fluorescence microscopy or flow cytometry. Applications include evaluating metabolic dysfunction in sepsis, where neutrophils exhibit hyperactive oxidative bursts ([35](https://doi.org/10.1631/jzus.B2101075)).

**5.3.2. Phagocytosis Assays**

Phagocytosis assays evaluate neutrophil capacity to internalize pathogens, linking metabolic activity to antimicrobial function. The antibody-dependent neutrophil phagocytosis (ADNP) assay uses flow cytometry to quantify immune complex uptake. Biotinylated antigens (e.g., HIV-1 gp120) are coated onto fluorescent NeutrAvidin beads, incubated with antibodies to form immune complexes, then exposed to neutrophils. Phagocytosis is measured as bead internalization via flow cytometry, with a "phagoscore" combining uptake frequency and intensity. This assay requires optimization of antigen density, antibody concentration, and neutrophil-to-bead ratios to minimize false positives (36,37). Alternatively, bacterial phagocytosis assays employ pH-sensitive fluorophores (e.g., pHrodo™) conjugated to *E. coli*; fluorescence intensifies upon phagolysosome acidification, allowing real-time kinetic tracking ([34,38](https://www.criver.com/products-services/discovery-services/pharmacology-studies/inflammation-autoimmune-studies/immunology/neutrophil-assays)). These assays identify defects in NADPH oxidase activity or granule fusion, as seen in chronic granulomatous disease ([31](https://www.nature.com/articles/s41392-024-02049-y)).

**5.3.3. NETosis Assays**

NETosis assays quantify neutrophil extracellular trap (NET) formation, a metabolic-dependent cell death mechanism. The IncuCyte ZOOM platform uses real-time imaging with membrane-permeant (Sytox Green) and impermeant (Hoechst 33342) DNA dyes to distinguish NETosis from apoptosis or necrosis. Neutrophils undergoing NETosis show nuclear decondensation followed by membrane rupture, detected via increased Sytox Green signal. This automated method applies size and intensity filters to quantify NETosis rates across stimuli (e.g., PMA, bacteria) and inhibitors. Validated against immunofluorescence microscopy, it reveals that NETosis requires glycolytic ATP production and reactive oxygen species (39,31). Microfluidic devices simultaneously analyze NETs and ROS in primary neutrophils using minimal blood volumes, enabling subset-specific profiling (e.g., low-density vs. normal-density neutrophils) ([34,40](https://academic.oup.com/ib/article/8/2/243/5115786?login=false)). Applications include studying dysregulated NETosis in autoimmune diseases like lupus ([39](https://pubmed.ncbi.nlm.nih.gov/29196457/)).

**5.3.4. Chemotaxis Assays**

Chemotaxis assays measure directed neutrophil migration toward chemoattractants, reflecting metabolic adaptability. The Boyden chamber/Transwell assay uses 5.0-μm porous membranes; neutrophils are seeded in serum-free medium in the upper chamber, while chemoattractants (e.g., IL-8/CXCL8) are added below. Migrated cells are quantified after 1 hour via ATP luminescence (CellTiter-Glo®). This assay captures PI3K/Akt and MAPK pathway dependencies, which regulate actin polymerization and metabolic reprogramming (41,42). Actin polymerization assays complement chemotaxis studies: neutrophils stimulated with fMLF or CXCL8 are fixed, permeabilized, and stained with Alexa Fluor 555-phalloidin to quantify F-actin via flow cytometry ([43](https://jlb.onlinelibrary.wiley.com/doi/pdf/10.1002/JLB.3AB0220-470R#:~:text=Actin%20polymerization%20assay,were%20analyzed%20by%20flow%20cytometry)). Reduced chemotaxis correlates with impaired glucose metabolism or mitochondrial dysfunction, as observed in sepsis or diabetes (44).

**5.3.5. Degranulation Assays**

Degranulation assays evaluate the release of cytotoxic granules, a process fueled by glycolytic ATP. Myeloperoxidase (MPO) and lactoferrin ELISAs quantify primary and secondary granule release in neutrophil supernatants. Neutrophils are stimulated with immune complexes (e.g., antibody-coated beads) or soluble agonists (e.g., fMLP), followed by supernatant collection at timed intervals. Degranulation kinetics reveal metabolic dependencies, as granule exocytosis requires glucose-driven ATP synthesis. This assay is crucial for diagnosing neutrophil-specific disorders like PAAND (Pyoderma Gangrenosum, Acne, and Neutrophilic Dermatosis), where aberrant degranulation drives autoinflammation ([45](https://www.ncbi.nlm.nih.gov/books/NBK482223/)). Applications include drug screening for inhibitors of pathological granule release (46,47).

**5.3.6. Cytokine Release Profiling**

Cytokine release profiling in neutrophils, particularly through advanced multiplex technologies like Luminex bead-based assays, has become an essential tool for analysing the metabolic underpinnings of neutrophil immunomodulatory functions. Upon encountering immune complexes, neutrophils rapidly shift their metabolism toward glycolysis, a process necessary for the robust production and secretion of cytokines such as TNF-α, G-CSF, and IL-1RA. This metabolic reprogramming is confirmed by the application of glycolytic inhibitors like 2-deoxyglucose (2-DG), which significantly suppress cytokine output, highlighting the dependence of cytokine synthesis on glucose metabolism. The Luminex platform enables simultaneous quantification of multiple cytokines from small sample volumes, allowing researchers to capture the dynamic and complex cytokine milieu produced by neutrophils during immune complex-driven activation. This approach has revealed that antibody-mediated phagocytosis not only facilitates pathogen clearance but also acts as a potent trigger for metabolic and functional reprogramming that occurs via activation of Fc receptors, which rapidly increase glycolysis to meet the higher energy demands of engulfing pathogens. This metabolic shift not only fuels the physical process of phagocytosis but also supports the production of inflammatory cytokines and the oxidative burst needed to kill microbes. In this way, antibody-mediated phagocytosis directly links neutrophil energy metabolism to a broader view of inflammatory immune responses(48,31). As a result, cytokine release profiling is now widely applied in studies of sepsis, autoimmune disease, and vaccine research to dissect neutrophil dysfunction, identify metabolic vulnerabilities, and guide the development of targeted immunotherapies ([49](https://pubmed.ncbi.nlm.nih.gov/34114369/)).

**5.3.7. Comprehensive Screening Approaches**

The NeutroFun Screen integrates multiple assays (viability, ROS, migration, phagocytosis) using a single neutrophil isolation. This 4-hour protocol employs real-time migration (xCELLigence), yeast phagocytosis on glass slides, and NBT reduction for ROS, followed by panoptic staining for preliminary NET detection. Designed for resource-limited settings, it balances speed and comprehensiveness while preserving cells for omics studies. Applications include rapid phenotyping in sepsis or drug development, where simultaneous functional readouts reveal interconnected metabolic dysregulations (50-52).

**5.3.8. Integration and Applications**

The integration of functional assays collectively maps neutrophil functions to their underlying metabolic states. For instance, the coupling between ROS production and phagocytosis is evident as NADPH oxidase-derived ROS optimizes phagosomal pH for pathogen killing (53). In NETosis, glycolysis fuels chromatin decondensation while the pentose phosphate pathway supports ROS generation (54). Chemotaxis relies on glycolytic energetics, with migrating neutrophils switching to glycolysis for rapid ATP generation (55). Importantly, standardization of neutrophil purification methods is critical because techniques such as immunomagnetic separation versus density-gradient centrifugation significantly alter functional readouts.

**5.4. Multi-Omics Integration and predictive modeling**

Last but not the least, multi-omics integration and predictive modeling represent a transformative approach in the study of neutrophil metabolism, enabling researchers to comprehensively profile and interpret the complex molecular networks that govern neutrophil function in health and disease. Multi-omics refers to the simultaneous analysis and integration of various layers of biological information, including genomics, transcriptomics, proteomics, metabolomics, and lipidomics, to provide a holistic view of cellular states and regulatory mechanisms. In neutrophil research, this approach has allowed for the identification of novel subtypes, functional states, and regulatory pathways that would be difficult to uncover using single-modality analyses.

When it comes to disease and disorder research, multi-omics approaches have revolutionized research on septic shock by enabling the simultaneous integration of “-omics” data to study the complex molecular networks underlying this life-threatening condition. By combining these diverse data layers, researchers can identify novel biomarkers, stratify patients into clinically meaningful subgroups, and uncover key regulatory pathways that drive disease progression and organ dysfunction in septic shock ([56](https://pmc.ncbi.nlm.nih.gov/articles/PMC9300837/)). For example, time-dependent multi-omics integration has revealed dynamic changes in immune signaling pathways, such as the Toll-like receptor 4 (TLR4) axis, that are not apparent in single-omics analyses, providing deeper insight into the pathophysiology of sepsis and its complications ([57](https://doi.org/10.1016/j.gpb.2023.04.002)). Multi-omics studies have also facilitated the discovery of proteomic and transcriptomic signatures that predict patient responses to therapies, such as fluid management strategies, and have highlighted the heterogeneity of septic shock by identifying distinct molecular endotypes associated with different clinical outcomes ([58](https://www.scientificarchives.com/article/identification-of-septic-shock-subgroups-for-fluid-strategy-formulation-a-multi-omics-integrated-approach)). Ultimately, the use of multi-omics in septic shock research is paving the way for precision medicine, allowing for more accurate diagnosis, risk stratification, and the development of targeted therapeutic interventions tailored to individual patient profiles ([59,6](https://pmc.ncbi.nlm.nih.gov/articles/PMC7088921/)(https:/www.frontiersin.org/journals/immunology/articles/10.3389/fimmu.2022.905601/full)0).

Moreover, multi-omics integration coupled with artificial intelligence (AI) and machine learning has enabled the discovery of new neutrophil clusters and the identification of hub genes that serve as potential biomarkers and therapeutic targets, particularly in complex conditions such as sepsis. In one study, single-cell RNA sequencing data from sepsis patients were analyzed alongside weighted gene co-expression network analysis (WGCNA) and multiple machine learning algorithms to pinpoint key gene modules and transcription factors regulating neutrophil responses. This integrative approach not only uncovered novel neutrophil subtypes enriched during sepsis but also validated hub genes in both human and mouse models, demonstrating the robustness and translational potential of predictive modeling in neutrophil research (61). Multi-omics integration has also been applied to explore the heterogeneity of NETosis in cancer and chronic inflammatory diseases. By combining transcriptomic, proteomic, and metabolic data, researchers have uncovered distinct metabolic reprogramming events-such as upregulation of glycolysis, oxidative phosphorylation, and hypoxia pathways-in NET-positive neutrophils. These findings illustrate how multi-omics can dissect the metabolic underpinnings of specific functional phenotypes, such as NETosis, and reveal regulatory mechanisms like mTOR-mediated autophagy inhibition (62). The integration of multi-omics data is increasingly supported by advanced computational tools and predictive modeling frameworks, which enable the synthesis of large, complex datasets into actionable biological insights. These methods facilitate the identification of key regulatory nodes, the mapping of metabolic pathways to neutrophil effector functions, and the prediction of disease-associated phenotypes. As such, multi-omics integration and predictive modeling are essential for unraveling the systems-level regulation of neutrophil immunometabolism and for guiding the development of targeted interventions in immune-mediated diseases (63,64).

References

1. Riffelmacher T, Clarke A, Richter FC, Stranks A, Pandey S, Danielli S, Hublitz P, Yu Z, Johnson E, Schwerd T, et al. Autophagy-Dependent Generation of Free Fatty Acids Is Critical for Normal Neutrophil Differentiation. *Immunity* (2017) 47:466-480.e5. doi: 10.1016/j.immuni.2017.08.005
2. Pandey S. Advances in metabolomics in critically ill patients with sepsis and septic shock. *Clin Exp Emerg Med* (2024) 12:4–15. doi: 10.15441/ceem.24.211
3. Mohammadnezhad L, Shekarkar Azgomi M, La Manna MP, Sireci G, Rizzo C, Badami GD, Tamburini B, Dieli F, Guggino G, Caccamo N. Metabolic Reprogramming of Innate Immune Cells as a Possible Source of New Therapeutic Approaches in Autoimmunity. *Cells* (2022) 11:1663. doi: 10.3390/cells11101663
4. Cosgrove J, Marçais A, Hartmann FJ, Bergthaler A, Zanoni I, Corrado M, Perié L, Cabezas-Wallscheid N, Bousso P, Alexandrov T, et al. A call for accessible tools to unlock single-cell immunometabolism research. *Nat Metab* (2024) 6:779–782. doi: 10.1038/s42255-024-01031-w.
5. Jeelani I, Nawaz A, Asif HM, Ahmad I, Gattu AK. Editorial: Recent advances in immunometabolism. *Front Pharmacol* (2024) 15: doi: 10.3389/fphar.2024.1422816
6. De Falco B, Giannino F, Carteni F, Mazzoleni S, Kim D-H. Metabolic flux analysis: a comprehensive review on sample preparation, analytical techniques, data analysis, computational modelling, and main application areas. *RSC Adv* (2022) 12:25528–25548. doi: 10.1039/D2RA03326G
7. Jin ES, Lee MH, Malloy CR. ^13^ C NMR of glutamate for monitoring the pentose phosphate pathway in myocardium. *NMR in Biomedicine* (2021) 34:e4533. doi: 10.1002/nbm.4533
8. Young JD. INCA: a computational platform for isotopically non-stationary metabolic flux analysis. *Bioinformatics* (2014) 30:1333–1335. doi: 10.1093/bioinformatics/btu015
9. He L, Wu SG, Zhang M, Chen Y, Tang YJ. WUFlux: an open-source platform for 13C metabolic flux analysis of bacterial metabolism. *BMC Bioinformatics* (2016) 17:444. doi: 10.1186/s12859-016-1314-0
10. Gupta S, Kaplan MJ. The role of neutrophils and NETosis in autoimmune and renal diseases. *Nat Rev Nephrol* (2016) 12:402–413. doi: 10.1038/nrneph.2016.71
11. Yao R, Li J, Feng L, Zhang X, Hu H. 13C metabolic flux analysis-guided metabolic engineering of Escherichia coli for improved acetol production from glycerol. *Biotechnol Biofuels* (2019) 12:29. doi: 10.1186/s13068-019-1372-4
12. Baysoy A, Bai Z, Satija R, Fan R. The technological landscape and applications of single-cell multi-omics. *Nat Rev Mol Cell Biol* (2023) 24:695–713. doi: 10.1038/s41580-023-00615-w
13. Wang Y-H, Wang L, Ho P-C. Decoding immunometabolism with next-generation tools: lessons from dendritic cells and T cells. *EMBO J* (2025) 44:5924–5939. doi: 10.1038/s44318-025-00569-z
14. Liu Z, Zhang Z, Zhang Y, Zhou W, Zhang X, Peng C, Ji T, Zou X, Zhang Z, Ren Z. Spatial transcriptomics reveals that metabolic characteristics define the tumor immunosuppression microenvironment via iCAF transformation in oral squamous cell carcinoma. *Int J Oral Sci* (2024) 16:9. doi: 10.1038/s41368-023-00267-8
15. Ahl PJ, Hopkins RA, Xiang WW, Au B, Kaliaperumal N, Fairhurst A-M, Connolly JE. Met-Flow, a strategy for single-cell metabolic analysis highlights dynamic changes in immune subpopulations. *Commun Biol* (2020) 3:305. doi: 10.1038/s42003-020-1027-9
16. Zhang S, Kong X, Yao M, Qi J, Li Y, Liang H, Zhou Y. Met-Flow analyses of the metabolic heterogeneity associated with different stages of cord blood-derived hematopoietic cell differentiation. *Front Immunol* (2024) 15: doi: 10.3389/fimmu.2024.1425585
17. Andrews JT, Zhang Z, Prasad GVRK, Huey F, Nazarova EV, Wang J, Ranaraja A, Weinkopff T, Li L-X, Mu S, et al. Metabolically active neutrophils represent a permissive niche for Mycobacterium tuberculosis. *Mucosal Immunol* (2024) 17:825–842. doi: 10.1016/j.mucimm.2024.05.007
18. Eriksson O, Håkansson LD, Karawajczyk M, Garwicz D. Neutrophil CD64 expression – comparison of two different flow cytometry protocols on EPICs MCL and the Leuko64^TM^ assay on a Celldyn Sapphire haematology analyser.*Scandinavian Journal of Clinical and Laboratory Investigation* (2015) 75:428–433. https://api.semanticscholar.org/CorpusID:8362532
19. Datta R, Miskolci V, Gallego-López GM, Britt E, Gillette A, Kralovec A, Giese MA, Qian T, Votava J, Zhao W, et al. Single cell autofluorescence imaging reveals immediate metabolic shifts of neutrophils with activation across biological systems. *Front Immunol* (2025) 16:1617993. doi: 10.3389/fimmu.2025.1617993
20. Grudzinska FS, Jasper A, Sapey E, Thickett DR, Mauro C, Scott A, Barlow J. Real-time assessment of neutrophil metabolism and oxidative burst using extracellular flux analysis. *Front Immunol* (2023) 14:1083072. doi: 10.3389/fimmu.2023.1083072
21. Walsh AJ, Cook RS, Manning HC, Hicks DJ, Lafontant A, Arteaga CL, Skala MC. Optical Metabolic Imaging Identifies Glycolytic Levels, Subtypes, and Early-Treatment Response in Breast Cancer. *Cancer Research* (2013) 73:6164–6174. doi: 10.1158/0008-5472.CAN-13-0527
22. Rodríguez‐Espinosa O, Rojas‐Espinosa O, Moreno‐Altamirano MMB, López‐Villegas EO, Sánchez‐García FJ. Metabolic requirements for neutrophil extracellular traps formation. *Immunology* (2015) 145:213–224. doi: 10.1111/imm.12437
23. Plitzko B, Loesgen S. Measurement of Oxygen Consumption Rate (OCR) and Extracellular Acidification Rate (ECAR) in Culture Cells for Assessment of the Energy Metabolism. *BIO-PROTOCOL* (2018) 8: doi: 10.21769/BioProtoc.2850
24. Agilent Seahorse XF. Glycolysis Stress Test Kit. User Guide. 2019.
25. Vijayaragavan K, Cannon BJ, Tebaykin D, Bossé M, Baranski A, Oliveria JP, Bukhari SA, Mrdjen D, Corces MR, McCaffrey EF, et al. Single-cell spatial proteomic imaging for human neuropathology. *acta neuropathol commun* (2022) 10:158. doi: 10.1186/s40478-022-01465-x
26. Keren L, Bosse M, Thompson S, Risom T, Vijayaragavan K, McCaffrey E, Marquez D, Angoshtari R, Greenwald NF, Fienberg H, et al. MIBI-TOF: A multiplexed imaging platform relates cellular phenotypes and tissue structure. *Sci Adv* (2019) 5:eaax5851. doi: 10.1126/sciadv.aax5851
27. Oliveira MF de, Romero JP, Chung M, Williams SR, Gottscho AD, Gupta A, Pilipauskas SE, Mohabbat S, Raman N, Sukovich DJ, et al. High-definition spatial transcriptomic profiling of immune cell populations in colorectal cancer. *Nat Genet* (2025) 57:1512–1523. doi: 10.1038/s41588-025-02193-3
28. Jing S, Wang H, Lin P, Yuan J, Tang Z, Li H. Quantifying and interpreting biologically meaningful spatial signatures within tumor microenvironments. *npj Precis Onc* (2025) 9:68. doi: 10.1038/s41698-025-00857-1 Brown EP, Dowell KG, Boesch AW, et al. Multiplexed Fc array for evaluation of antigen-specific antibody effector profiles. Journal of immunological methods. 2017 Apr 1;443:33-44.
29. Nunes JB, Ijsselsteijn ME, Abdelaal T, Ursem R, Van Der Ploeg M, Giera M, Everts B, Mahfouz A, Heijs B, De Miranda NFCC. Integration of mass cytometry and mass spectrometry imaging for spatially resolved single-cell metabolic profiling. *Nat Methods* (2024) 21:1796–1800. doi: 10.1038/s41592-024-02392-6
30. Zhang F, Xia Y, Su J, Quan F, Zhou H, Li Q, Feng Q, Lin C, Wang D, Jiang Z. Neutrophil diversity and function in health and disease. *Sig Transduct Target Ther* (2024) 9:343. doi: 10.1038/s41392-024-02049-y
31. Blanter M, Cambier S, De Bondt M, Vanbrabant L, Pörtner N, Abouelasrar Salama S, Metzemaekers M, Marques PE, Struyf S, Proost P, et al. Method Matters: Effect of Purification Technology on Neutrophil Phenotype and Function. *Front Immunol* (2022) 13:820058. doi: 10.3389/fimmu.2022.820058
32. Chen Y, Junger WG. “Measurement of Oxidative Burst in Neutrophils.,” In: Ashman RB, editor. *Leucocytes*. Totowa, NJ: Humana Press (2012). p. 115–124 doi: 10.1007/978-1-61779-527-5_8
33. Moussavi-Harami SF, Mladinich KM, Sackmann EK, Shelef MA, Starnes TW, Guckenberger DJ, Huttenlocher A, Beebe DJ. Microfluidic device for simultaneous analysis of neutrophil extracellular traps and production of reactive oxygen species. *Integr Biol* (2016) 8:243–252. doi: 10.1039/C5IB00225G
34. Lu J, Liu J, Li A. Roles of neutrophil reactive oxygen species (ROS) generation in organ function impairment in sepsis. *J Zhejiang Univ Sci B* (2022) 23:437–450. doi: 10.1631/jzus.B2101075
35. Karsten CB, Mehta N, Shin SA, Diefenbach TJ, Slein MD, Karpinski W, Irvine EB, Broge T, Suscovich TJ, Alter G. A versatile high-throughput assay to characterize antibody-mediated neutrophil phagocytosis. *J Immunol Methods* (2019) 471:46–56. doi: 10.1016/j.jim.2019.05.006
36. Kuhns DB, Priel DAL, Chu J, Zarember KA. Isolation and Functional Analysis of Human Neutrophils. *CP in Immunology* (2015) 111: doi: 10.1002/0471142735.im0723s111
37. Rodriguez-Moncayo R, Pons S, Tavares LP, Jeon H, Preuss J-A, Bahnemann J, Han J, Levy BD, Voldman J. Inertial Microfluidics Enables Functional Analysis of Neutrophils Isolated from Ultralow Blood Volume Samples. *Anal Chem* (2025) 97:8419–8428. doi: 10.1021/acs.analchem.5c00102
38. Lika J, Fan J. Carbohydrate metabolism in supporting and regulating neutrophil effector functions. *Curr Opin Immunol* (2024) 91:102497. doi: 10.1016/j.coi.2024.102497
39. Chicca IJ, Milward MR, Chapple ILC, Griffiths G, Benson R, Dietrich T, Cooper PR. Development and Application of High-Content Biological Screening for Modulators of NET Production. *Front Immunol* (2018) 9:337. doi: 10.3389/fimmu.2018.00337
40. Nuzzi PA, Lokuta MA, Huttenlocher A. “Analysis of Neutrophil Chemotaxis.,” In: Coutts AS, editor. *Adhesion Protein Protocols*. Totowa, NJ: Humana Press (2007). p. 23–35 doi: 10.1007/978-1-59745-353-0_3 Papayannopoulos V. Neutrophil extracellular traps in immunity and disease. Nature Reviews Immunology. 2018 Feb;18(2):134-47.
41. Hoang AN, Jones CN, Dimisko L, Hamza B, Martel J, Kojic N, Irimia D. Measuring neutrophil speed and directionality during chemotaxis, directly from a droplet of whole blood. *Technology* (2013) 01:49–57. doi: 10.1142/S2339547813500040
42. Metzemaekers M, Vandendriessche S, Berghmans N, Gouwy M, Proost P. Truncation of CXCL8 to CXCL8(9-77) enhances actin polymerization and in vivo migration of neutrophils. *Journal of Leukocyte Biology* (2020) 107:1167–1173. doi: 10.1002/JLB.3AB0220-470R
43. Frydrych LM, Fattahi F, He K, Ward PA, Delano MJ. Diabetes and Sepsis: Risk, Recurrence, and Ruination. *Front Endocrinol (Lausanne)* (2017) 8:271. doi: 10.3389/fendo.2017.00271
44. Schmieder SJ, Krishnamurthy K. “Pyoderma Gangrenosum.,” *StatPearls*. Treasure Island (FL): StatPearls Publishing (2025) http://www.ncbi.nlm.nih.gov/books/NBK482223/ [Accessed February 19, 2026]
45. Soehnlein O, Weber C, Lindbom L. Neutrophil granule proteins tune monocytic cell function. *Trends in Immunology* (2009) 30:538–546. doi: 10.1016/j.it.2009.06.006
46. Bedouhène S, Dang PM-C, Hurtado-Nedelec M, El-Benna J. “Neutrophil Degranulation of Azurophil and Specific Granules.,” In: Quinn MT, DeLeo FR, editors. *Neutrophil: Methods and Protocols*. New York, NY: Springer US (2020). p. 215–222 doi: 10.1007/978-1-0716-0154-9_16
47. Liu C, Chu D, Kalantar‐Zadeh K, George J, Young HA, Liu G. Cytokines: From Clinical Significance to Quantification. *Advanced Science* (2021) 8:2004433. doi: 10.1002/advs.202004433
48. Vella R, Panci D, Carini F, Malta G, Vieni S, David S, Albano GD, Puntarello M, Zerbo S, Argo A. Cytokines in sepsis: a critical review of the literature on systemic inflammation and multiple organ dysfunction. *Front Immunol* (2025) 16:1682306. doi: 10.3389/fimmu.2025.1682306
49. Luz IS, Takaya R, Ribeiro DG, Silva NS, Fontes L, Castro MS, Fontes W. A Set of Screening Techniques for a Quick Overview of the Neutrophil Function. *Journal of Visualized Experiments (JoVE)* (2024)e65329. doi: 10.3791/65329
50. Gierlikowska B, Stachura A, Gierlikowski W, Demkow U. Phagocytosis, Degranulation and Extracellular Traps Release by Neutrophils-The Current Knowledge, Pharmacological Modulation and Future Prospects. *Front Pharmacol* (2021) 12:666732. doi: 10.3389/fphar.2021.666732
51. Timmer KD, Floyd DJ, Scherer AK, Crossen AJ, Atallah J, Viens AL, Sykes DB, Mansour MK. Multiparametric Profiling of Neutrophil Function via a High-Throughput Flow Cytometry-Based Assay. *Cells* (2023) 12:743. doi: 10.3390/cells12050743
52. Nguyen GT, Green ER, Mecsas J. Neutrophils to the ROScue: Mechanisms of NADPH Oxidase Activation and Bacterial Resistance. *Front Cell Infect Microbiol* (2017) 7: doi: 10.3389/fcimb.2017.00373
53. Azzouz D, Palaniyar N. How Do ROS Induce NETosis? Oxidative DNA Damage, DNA Repair, and Chromatin Decondensation. *Biomolecules* (2024) 14:1307. doi: 10.3390/biom14101307
54. Jeon J-H, Hong C-W, Kim EY, Lee JM. Current Understanding on the Metabolism of Neutrophils. *Immune Netw* (2020) 20:e46. doi: 10.4110/in.2020.20.e46
55. Qiao J, Cui L. Multi-Omics Techniques Make it Possible to Analyze Sepsis-Associated Acute Kidney Injury Comprehensively. *Front Immunol* (2022) 13:905601. doi: 10.3389/fimmu.2022.905601
56. Na A-Y, Lee H, Min EK, Paudel S, Choi SY, Sim H, Liu K-H, Kim K-T, Bae J-S, Lee S. Novel Time-Dependent Multi-Omics Integration in Sepsis-Associated Liver Dysfunction. *Genomics, Proteomics & Bioinformatics* (2023) 21:1101–1116. doi: 10.1016/j.gpb.2023.04.002
57. Lei X, Zhang Z. Identification of Septic Shock Subgroups for Fluid Strategy Formulation: A Multi-Omics Integrated Approach. *J Cell Immunol* (2025) 7:49–51. doi: 10.33696/immunology.7.224
58. Liu X, Ren H, Peng D. Sepsis biomarkers: an omics perspective. *Front Med* (2014) 8:58–67. doi: 10.1007/s11684-014-0318-2
59. Jin X, Shen H, Zhou P, Yang J, Yang S, Ni H, Yu Y, Zhang Z. Research Progress on Sepsis Diagnosis and Monitoring Based on Omics Technologies: A Review. *Diagnostics (Basel)* (2025) 15:2887. doi: 10.3390/diagnostics15222887
60. Xu P, Tao Z, Zhang C. Integrated multi-omics and artificial intelligence to explore new neutrophils clusters and potential biomarkers in sepsis with experimental validation. *Front Immunol* (2024) 15:1377817. doi: 10.3389/fimmu.2024.1377817
61. Fu Y, Tao J, Gu Y, Liu Y, Qiu J, Su D, Wang R, Luo W, Liu T, Zhang F, et al. Multiomics integration reveals NETosis heterogeneity and TLR2 as a prognostic biomarker in pancreatic cancer. *npj Precis Onc* (2024) 8:109. doi: 10.1038/s41698-024-00586-x
62. Sibilio P, De Smaele E, Paci P, Conte F. Integrating multi-omics data: Methods and applications in human complex diseases. *Biotechnol Rep (Amst)* (2025) 48:e00938. doi: 10.1016/j.btre.2025.e00938
63. Qin D, Zheng Y, Wang L, Lin Z, Yao Y, Fei W, Zheng C. Unraveling shared diagnostic genes and cellular microenvironmental changes in endometriosis and recurrent implantation failure through multi-omics analysis. *Sci Rep* (2025) 15:9110. doi: 10.1038/s41598-025-93146-7
